# Detection of Growth-Related Quantitative Trait Loci and High-Resolution Genetic Linkage Maps Using Simple Sequence Repeat Markers in the Kelp Grouper (*Epinephelus bruneus*)

**DOI:** 10.1007/s10126-015-9673-5

**Published:** 2015-10-28

**Authors:** Kanonkporn Kessuwan, Satoshi Kubota, Qi Liu, Motohiko Sano, Nobuaki Okamoto, Takashi Sakamoto, Hirofumi Yamashita, Yoji Nakamura, Akiyuki Ozaki

**Affiliations:** Faculty of Marine Science, Tokyo University of Marine Science and Technology, 4-5-7 Konan, Minato-ku, Tokyo, 108-8477 Japan; Ehime Research Institute of Agriculture, Forestry and Fisheries, Fisheries Research Center, 5516 Shitaba, Uwajima-shi, Ehime, 798-0104 Japan; National Research Institute of Fisheries Science, Fisheries Research Agency, 2-12-4 Fukuura, Kanazawa-ku, Yokohama, Kanagawa 236-8648 Japan; National Research Institute of Aquaculture, Fisheries Research Agency, 422-1 Nakatsuhamaura, Minamiise-cho, Watarai-gun Mie, 516-0193 Japan; Department of Fisheries, Coastal Fisheries Research and Development Bureau, 50 Kaset Klang Jatujak, Bangkok, 10900 Thailand; Center for Marine Ranching Engineering Science Research of Liaoning, Dalian Ocean University, 52 Heishijiao Street, Dalian, 116023 China

**Keywords:** *Epinephelus bruneus*, Simple sequence repeat (SSR), High-resolution genetic linkage map, Quantitative trait loci (QTLs)

## Abstract

To initiate breeding programs for kelp grouper (*Epinephelus bruneus*), the establishment of genetic linkage maps becomes essential accompanied by the search for quantitative trait loci that may be utilized in selection programs. We constructed a high-resolution genetic linkage map using 1055 simple sequence repeat (SSR) markers in an F_1_ family. Genome-wide and chromosome-wide significances of growth-related quantitative trait loci (QTLs) (body weight (BW) and total length (TL)) were detected using non-parametric mapping, Kruskal-Wallis (K-W) analysis, simple interval mapping (IM) and a permutation test (PT). Two stages and two families of fish were used to confirm the QTL regions. Ultimately, 714 SSR markers were matched that evenly covered the 24 linkage groups. In total, 509 and 512 markers were localized to the female and male maps, respectively. The genome lengths were approximately 1475.95 and 1370.39 cM and covered 84.68 and 83.21 % of the genome, with an average interval of 4.1 and 4.0 cM, in females and males, respectively. One major QTL affecting BW and TL was found on linkage group EBR 17F that identified for 1 % of the genome-wide significance and accounted for 14.6–18.9 and 14.7–18.5 % of the phenotypic variance, and several putative QTL with 5 % chromosome-wide significance were detected on eight linkage groups. Furthermore, the confirmed results of the regions harboring the major and putative QTLs showed consistent significant experiment-wide values of 1 and 5 % as well as a chromosome-wide value of 5 %. We identified growth-related QTLs that could be applied to find candidate genes for growth traits in further studies, and potentially useful in MAS breeding.

**Electronic supplementary material:**

The online version of this article (doi:10.1007/s10126-015-9673-5) contains supplementary material, which is available to authorized users.

## Introduction

The kelp grouper (*Epinephelus bruneus*) is a commercially important marine fish in East Asia. This species belongs to the subfamily Ephinephelinae, family Serranidae, and order Perciformes. Groupers, or Serranidae in general, are protogynous, which means they first start life as a female fish and then later switch into being males once they pass a certain size threshold or due to social cues (Lee et al. 2002; Tsuchihashi et al. 2003; Yeh et al. 2003) and matures at more than 6 years of age (Liu et al. 2013). The kelp grouper is a carnivorous fish that feeds on small fish and crustaceans. Generally, juvenile kelp groupers are found in shallow water estuaries (Heemstra and Randall 1995) and coastal areas, while the adult fish inhabit waters ranging 20–200 m of depth (An et al. 2011) around coral reefs, rocky reefs, and mud bottom areas. The maximum size of a kelp grouper is reported to be about 128 cm in length and 33 kg in body weight (Tupper and Sheriff 2008). At least 16 species of grouper, including the kelp grouper, have been used successfully in aquaculture in many countries in East Asia (Tupper and Sheriff 2008).

In Japan, the kelp grouper has a high value because of its high market demand and low quantity of catch in natural waters (Mitcheson et al. 2003). Recently, this species was listed as a vulnerable species by the International Union for Conservation of Nature and Natural Resources (the IUCN Red List of Threatened Species) because of the rapid decrease in the natural population (Thierry et al. 2008). The kelp grouper is a target species for aquaculture in Japan (Fui et al. 2014); however, during artificial larval rearing, high mortality is frequent in the early life stages (Sawada et al. 1999). In addition, the kelp grouper grows slowly in farms and a prolonged farming period is required to reach a marketable size. To date, domestication of broodstock and a selective breeding program on a commercial scale for the kelp grouper in Japan have not yet been fully developed. Marker-assisted selection (MAS) based on quantitative trait loci (QTLs) is an effective method to improve quantitative traits (Max and Anatoly 2007) such as slow growth and high mortality in the larval stage of groupers.

In the recent decades, several genetic linkage maps of fin fish have been constructed using genetic markers, such as those for rainbow trout (*Oncorhynchus mykiss*), using simple sequence repeats (SSRs); Atlantic salmon (*Salmo salar*) using amplified fragment length polymorphisms (AFLPs) and SSRs; brown trout (*Salmo trutta*, AFLPs and SSRs); Nile tilapia (*Oreochromis niloticus*, AFLPs and SSRs); channel catfish (*Ictalurus punctatus*, AFLPs and SSRs), Japanese flounder (*Paralichthys olivaceus*, AFLPs and SSRs); ayu (*Plecoglossus altivelis*, AFLPs and SSRs; and yellowail (*Seriola quinqueradiata*, SSRs) (Danzmann and Ghabi 2007). A genetic linkage map of the kelp grouper was produced based on microsatellite markers (Liu et al. 2013). Several studies on growth-related quantitative traits (QTLs) have been carried out recently on fishes such as the rainbow trout, Nile tilapia, Arctic char (*Salvelinus alpinus*) (Danzmann and Ghabi 2007), Atlantic salmon (Baranski et al. 2010), barramundi (*Lates calcarifer*) (Wang et al. 2008), and turbot (*Scophthalmus maximus*) (Molano et al. 2011).

SSR markers are highly polymorphic and show high inheritance and codominance of inheritance, making them suitable to identify homozygotes and heterozygotes. They are usually evenly distributed throughout the genome, and their results are simple to interpret, highly reproducible, and easily automated (Liu 2007). Thus, they are useful to construct a genetic linkage map. Nevertheless, the detection of a reasonable proportion of QTLs segregating in a population requires a large number of markers to increase the accuracy of QTL detection.

Economic traits in aquaculture fish, especially growth-related quantitative traits, are the main goals for improvement in a genetic breeding program. Growth-related traits have been measured and reported in several economically important marine fishes (Yue 2013). To study growth-related traits in fish and other species by molecular tools is complex, because growth-related traits are influenced not only by genetics, but also by the environment (Abraham et al. 2007; Molano et al. 2011).

In 2013, the first-generation genetic linkage map for the kelp grouper was constructed using 222 microsatellite markers, covering 23 and 25 linkage groups in the male and female maps, with marker intervals of 5.0 and 6.7 cM, respectively (Liu et al. 2013). In the present study, a high-resolution genetic linkage map and a genome scan for QTLs affecting growth-related traits (BW and TL) in F_1_ progeny of kelp groupers were conducted. These results could be used to investigate candidate genes that will accelerate genetic improvement using MAS breeding programs in the kelp grouper.

## Materials and Methods

### Reference Family and DNA Extraction

Paternal half-sib F_1_ progeny from two families (families A and B) produced from two females and a single male were used. The fish were taken from recently derived wild broodstock of the kelp grouper maintained at the Ehime Fisheries Research Center, Japan. Fish were measured at two timepoints. Stage I at 5 months post-hatching (average total length of 150 mm), and stage II at 11 months post-hatching. At stage I, individual fish were tracked using an embedded passive integrated transponder (PIT) tag. This facilitated comparisons of fast growth phases that occur in the fish at this point in their development. All fish were measured for body weight (BW) and total length (TL). In total, 360 and 163 progeny in stages I and II of family A; and 112 and 45 progeny in stages I and II of family B were measured for BW and TL. Fin clip samples were collected and kept in absolute ethanol (99.9 % ethanol solution). DNA extraction was carried out from these samples using the Agincourt DNAdvance Genomic DNA Isolation Kit (Beckman Coulter, USA), following the manufacturer’s recommended protocol. The quality and quantity of the extracted DNA was quantified using a spectrophotometer (Untrospec 2100 pro, GE USA) and the DNA was diluted to 10 ng/μL for PCR.

The high-resolution genetic linkage maps to find candidate growth-related QTL regions were constructed by using the parents and 90 F_1_ progeny in stage II of family A. After that, all progeny in both stages of families A and B were used to confirm the candidate QTL regions.

### SSR Markers and Genotyping

A total of 2348 microsatellite-enriched segments from the kelp grouper were developed using next-generation sequencing (NGS) by the GS FLX system (Roche, Switzerland) (denoted as the EBR series) (Kubota et al. 2014) and 889 simple tandem repeats (STR) markers were obtained from the NCBI database of a cross section of species in the subfamily Epinephelinae (denoted as the STR series) (Chapman et al. 1999; Dong et al. 2008; Liu et al. 2008; Lo and Yue 2007; Mokhtar et al. 2011; Ramirez et al. 2006; Renshaw et al. 2010; Rivera et al. 2003; Zeng et al. 2008; Zhao et al. 2009a, b; Zhu et al. 2005). In total, 1867 SSR markers (1466 EBR markers and 401 STR markers) were designed using the TROLL program at http://wsmartins.net/websat/ (Martins et al. 2009) under the default settings and considering a product size of 100–250 bp. For the SSR markers, the forward primers were labeled with tetrachloro-6-carboxy-fluorescein (TET) fluorescent dye at the 5′-end. Polymerase chain reactions (PCR) were performed in 11 μl volumes containing 50 ng of genomic DNA, 1× Ex Taq buffer (Mg^2+^ free), 2.0 mM MgCl_2_, 0.2 mM dNTP, 1 % BSA, 0.025 U of Taq polymerase (Takara: Ex-Taq™ (Mg^2+^ free buffer)), 0.5 pmol/μL of the reverse primer, and 0.05 pmol/μL of the forward primer. Cycle amplification was performed on an MJ PTC-100 (Bio-Rad, USA), with the program conditions of 95 °C for 5 min for initial denaturation; followed by 36 cycles of 30 s at 95 °C, 1 min at the annealing temperature 56 °C, and 1 min at 72 °C, and a final extension at 72 °C for 10 min. The amplified products were mixed with an equal volume of loading buffer (98 % formaldehyde, 10 mM EDTA, and 0.05 % bromophenol blue), heated for 10 min at 95 °C and then immediately cooled on ice. The samples were separated on a 6 % polyacrylamide gel containing 7 M urea and 0.5× Trizma base/Boric Acid/EDTA-2Na (TBE) buffer and 40 % Page-plus (Amresco, USA) with a 500-bp DNA ladder (GeneScan™-500 TAMRA™). Electrophoresis was performed using 0.5× TBE buffer at a constant voltage of 1800 V for 1.5 h. After electrophoresis, the gel was scanned and imaged using an FMBIO III Multi-View fluorescence image analyzer (Hitachi-soft, Japan).

### Linkage Analysis

Linkage analysis was performed using LINKMFEX version 2.3 (Danzmann 2006). This application can separate alleles that originated from males or females. To avoid errors during genotyping, the accuracy of genotypes in their progeny was checked from parental male and female alleles. Genotype data were converted to a backcross format even though the grandparent genotype was unknown. Pairwise analysis was performed, and markers were sorted into linkage groups at a logarithm of odds (LOD) threshold of 4.0. Linkage phases were determined retrospectively by examining the assortment of alleles among linked markers. The goodness of fit of the chi-square analysis ( *χ*^2^) was used to test for Mendelian segregation distortion of the locus. Therefore, the distance of the marker was estimated on each linkage group, assuming the Kosambi mapping function. Double recombination was checked using the application in Map Manager QTX (Manly et al. 2001). Graphical representation of the linkage groups was performed using MAPCHART version 2.1 (Voorrips 2002). In addition, a consensus linkage map was constructed using JoinMap version 4 (Ooijen 2006) and the module of the combined group for map integration was used to integrate the sex-specific linkage maps.

### Estimation of Genome Size and Coverage

A sex-specific map of genome length was estimated by two different calculation methods. First, genome estimation size 1 (*G*_e1_) was calculated by adding 2*s*, where *s* is the average framework marker spacing that was calculated by dividing the summed length of all the genetic linkage groups by the number of intervals (number of markers minus the number of genetic linkage groups) to the length of each genetic linkage group, accounting for chromosome ends beyond the terminal markers coverage. Second, genome estimation size 2 (*G*_e2_) was calculated by multiplying the length of each genetic linkage group by a factor (*m* + 1)/(*m* − 1). Where *m* is the number of framework markers for each genetic linkage group (Chakravarti et al. 1990). The estimated genome length (*G*_e_) for each sex was used as an average of the two estimates (Fishman et al. 2001; Sanchez et al. 2010). The genome coverage for each sex was calculated as the observed genome length (*G*_oa_) divided by the estimated genome length (*G*_e)_ (Song et al. 2013) while the observed genome length (*G*_oa_) was taken as the combination of total length in all linkage group.

### QTL Analysis

First, the normality of the phenotypes (BW and TL) was tested using the Kolmogorov-Smirnov test (*N* > 50) and Shapiro-Wilk test (*N* < 50), implemented in SPSS 16.0 package. The data were converted to *Z* scores before analysis using MapQTL software.

QTL analysis was carried out using MapQTL 5 software (Ooijen 2004). Ninety F_1_ progeny from stage II of family A were used to find candidate QTLs. A non-parametric Kruskal-Wallis analysis was used to determine the significance level of all marker loci associated with the growth-related traits (BW and TL). Meanwhile, simple interval mapping was used to detect significant associations with growth-related traits and marker loci in the data sets under the significant threshold of genome-wide (*P* value < 0.01 and *P* value < 0.05) and chromosome-wide (*P* value < 0.05) analyses. A minimum LOD threshold of 4.0 was used for determining a significant QTL and the percentage of phenotypic variance of each QTL. Permutation tests were performed (1000 replicates) to determine the LOD threshold by type one error. The significant thresholds derived from the permutation tests was estimated by dividing the nominal *P* value by the total number of chromosomes (Churchill and Doerge 1994; Ozaki et al. 2013). A graphical representation of the significant QTLs was constructed using MAPCHART version 2.1 and MapQTL 5. The results of the growth-related QTL regions of stage II family A were confirmed to be reproducible in the other stage and family.

## Results

### Correlation of Phenotypes and Growth-Related Traits in Families A and B

The correlation of phenotypes was tested using Pearson’s correlation coefficient. The results showed a high correlation between BW and TL in both stages of the two families (Table 1). The normal distribution of the phenotype was tested by a Kolmogorov-Smirnov test or Shapiro-Wilk test depending on the number of samples (Table 2, Additional file 1). The high correlation between BW and TL and normal distribution of phenotypes in stage II of family A led us to select family A to construct the high genetic linkage map and to screen candidate QTL regions.

**Table 1:** Pearson correlation coefficients for total length and body weight

Family	Stage	No. of progeny		Total length	Body weight
A	I	360	Total length		0.729*
			Body weight	0.729*	
	II	163	Total length		0.968*
			Body weight	0.968*	
B	I	112	Total length		0.814*
			Body weight	0.814*	
	II	45	Total length		0.986*
			Body weight	0.986*	

*Correlation at 0.01 significance level (two-tailed)

**Table 2:** Phenotypic values of growth-related traits

Traits	Phenotypic and normal distribution			
	Family A		Family B	
	Stage I	Stage II	Stage I	Stage II
Number of progeny	360	163	112	45
Total length (mm)				
Maximum	164.00	271.00	156.00	258.00
Minimum	117.00	192.00	98.00	118.00
Average	143.81	228.25	139.55	219.02
STD	7.75	13.43	10.35	16.31
Kolmogorov-Smirnov	0.000	0.200*^a^	0.011	–
Shapiro-Wilk	–	–	–	0.358*
Body weight (g)				
Maximum	58.00	253.30	49.40	228.00
Minimum	17.60	93.00	17.00	89.40
Average	38.63	161.27	37.84	145.23
STD	6.38	27.96	7.75	31.60
Kolmogorov-Smirnov	0.200*^a^	0.200*^a^	0.053*	–
Shapiro-Wilk	–	–	–	0.515*

Kolmogorov-Smirnov (*N* > 50); Shapiro-Wilk (*N* < 50)

*P ≧ 0.05 normal distribution of phenotypic

^a^This is the lower bound of the rue significance

### High-Resolution Genetic Linkage Map and Genome Coverage

**Fig. 1:**
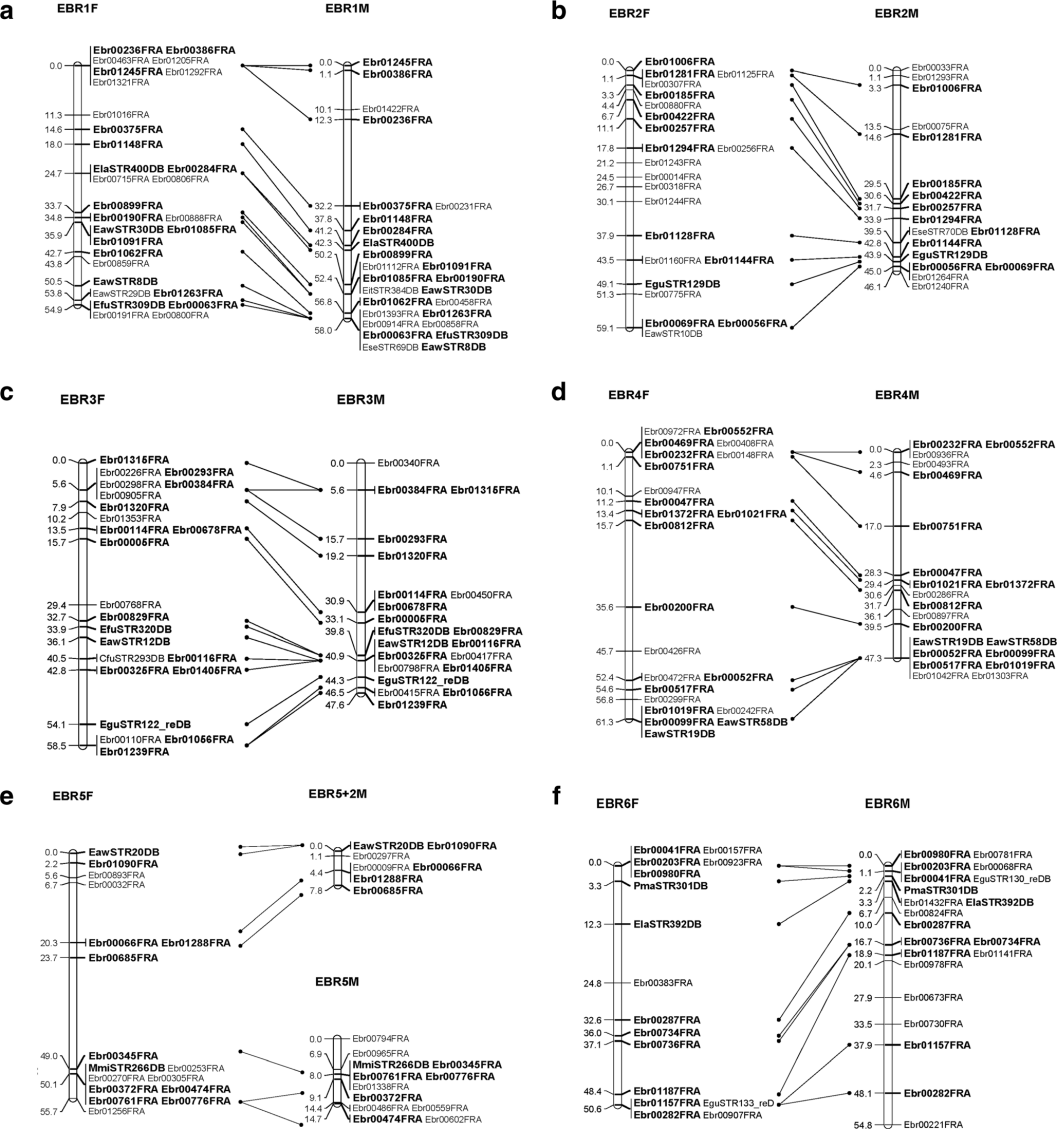

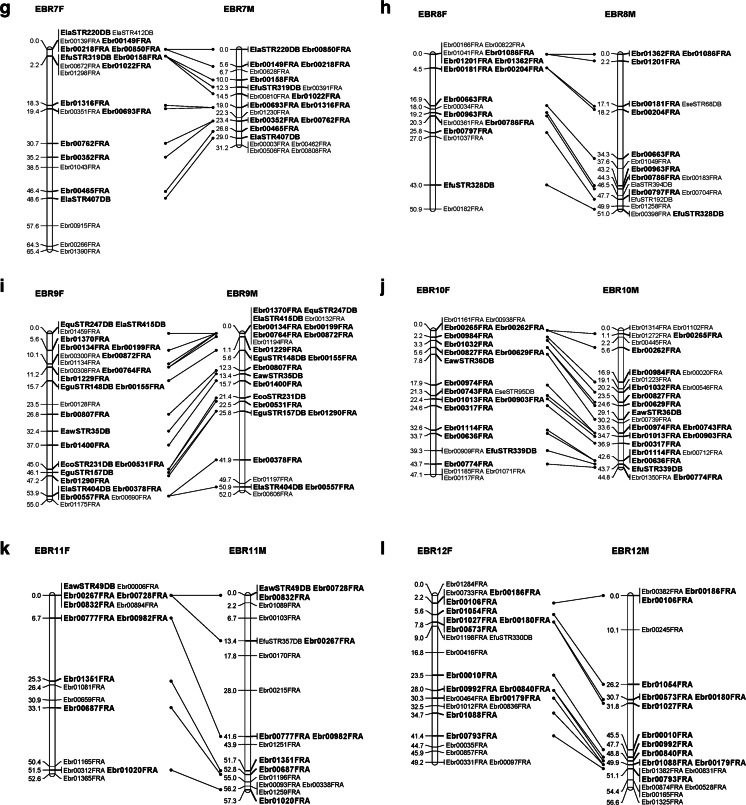

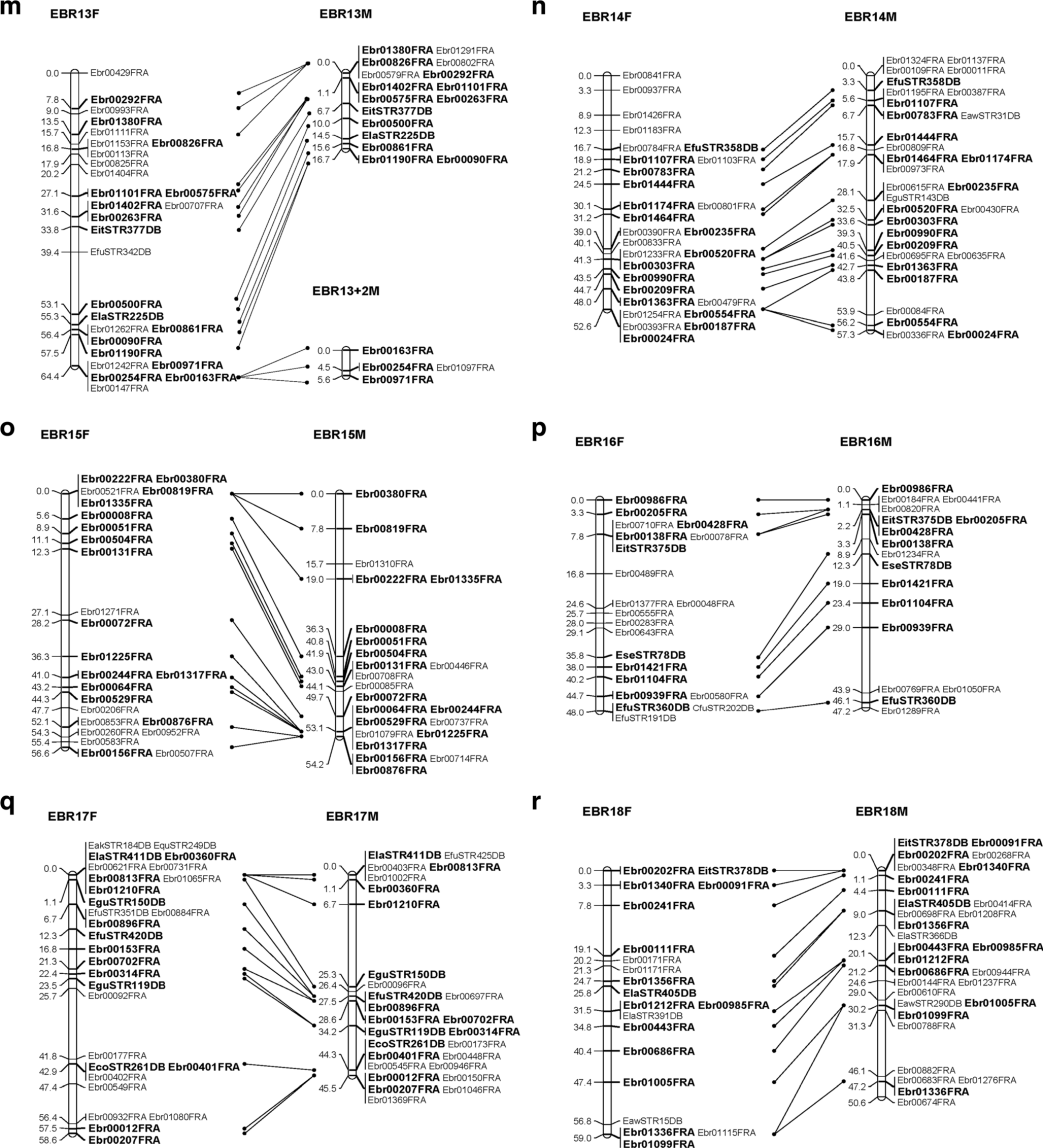

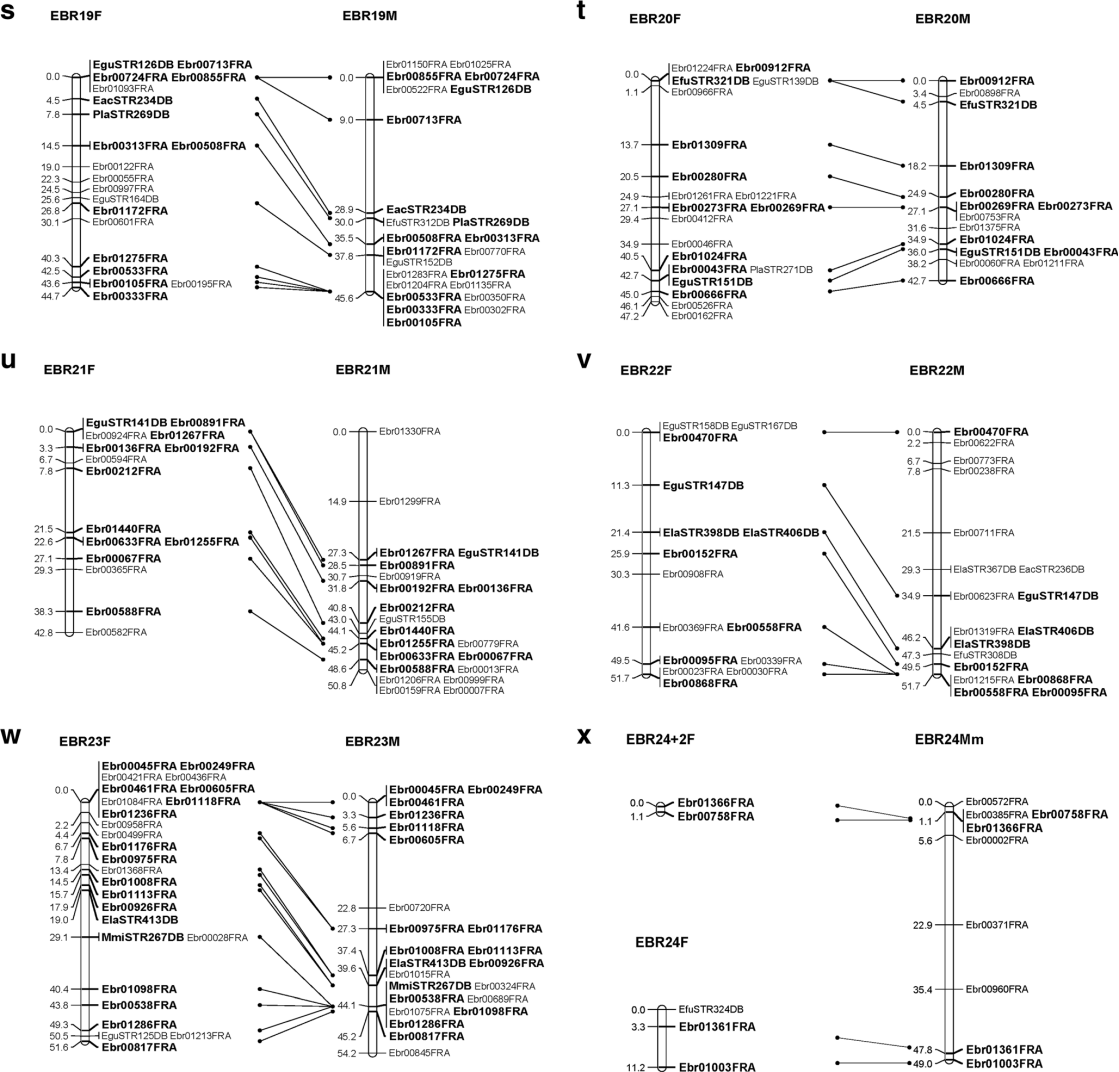
Kelp grouper female (*left*) and male (*right*) maps, linkage groups EBR 1–EBR 24. **a** EBR 1. **b** EBR 2. **c** EBR 3. **d** EBR 4. **e** EBR 5. **f** EBR 6. **g** EBR 7. **h** EBR 8. **i** EBR 9. **j** EBR 10. **k** EBR 11. **l** EBR 12. **m** EBR 13. **n** EBR 14. **o** EBR 15. **p** EBR 16. **q** EBR 17. **r** EBR 18. **s** EBR 19. **t** EBR 20. **u** EBR 21. **v** EBR 22. **w** EBR 23. **x** EBR 24. Total lengths of linkage groups are expressed in Kosambi cM. SSR markers are coded as “EBR” and “STR”. *Bold letters* indicate co-segregating microsatellite loci between the female and male maps

A total of 1867 SSR markers were designed. Of them, approximately 1050 SSR markers were polymorphic (56.2 %), and composed 905 EBR and 145 STR SSR markers. Ultimately, 714 SSR markers were used to construct a linkage map with reference species. The list of SSR markers used for mapping is given in additional file 2. Twenty-four genetic linkage groups (LG1–LG24) were identified. The female linkage map contained 509 markers distributed in 24 linkage groups (EBR 1F–EBR 24F) (Fig. 1). The total genome size of the female map was estimated as 1249.8 cM. The number of markers per linkage group varied from 5 to 29, with an average of 21; the longest linkage group of the female map extended to 65.4 cM (EBR 7F). Meanwhile, 512 markers were distributed in 24 linkage groups of the male map (EBR 1M–EBR 24M) (Fig. 1). The total genome was estimated at 1140.3 cM. The longest linkage group of the male extended to 58.0 cM (EBR 1), while the average number of markers per linkage group was 21, and varying from 9 to 31. The framework interval in each group was estimated based on the distance between clusters or markers, because some markers located on the same cluster. The female and male linkage maps comprised 305 and 285 framework, respectively, and the average interval between markers was 4.1 and 4.0 cM, respectively (Tables 3 and 4).

**Table 3:** Number of markers and information on the genetic linkage maps of the kelp grouper

	No. of marker	Female							LG	Male						
LG		No. of markers			Total length	Interval length	Genome length	Genome length		No. of markers			Total length	Interval length	Genome length	Genome length
		Map marker	Framework	Interval	cM	cM	*G*_e1_	*G*_e2_		Map marker	Framework	Interval	cM	cM	*G*_e1_	*G*_e2_
EBR 1F	38	29	13	12	54.90	4.22	63.80	64.05	EBR 1M	26	12	11	58.00	4.83	66.80	68.55
EBR 2F	28	22	16	15	59.10	3.69	68.00	66.98	EBR 2M	17	14	13	46.10	3.29	54.90	53.19
EBR 3F	29	23	14	13	58.50	4.18	67.40	67.5	EBR 3M	21	11	10	47.60	4.33	56.40	57.12
EBR 4F	29	23	12	11	61.30	5.11	70.20	72.45	EBR 4M	21	11	10	47.30	4.30	56.10	56.76
EBR 5F	25	17	9	8	55.70	6.19	64.60	69.63	EBR 5M	12	5	4	14.70	2.94	23.50	22.05
–									EBR 5 + 2M	7	4	3	7.80	1.95	16.60	13.00
EBR 6F	27	16	9	8	50.60	5.62	59.50	63.25	EBR 6M	21	14	13	54.80	3.91	63.60	63.23
EBR 7F	30	22	12	11	65.40	5.45	74.30	77.29	EBR 7M	21	12	11	31.20	2.60	40.00	36.87
EBR 8F	25	17	10	9	50.90	5.09	59.80	62.21	EBR 8M	18	12	11	51.00	4.25	59.80	60.27
EBR 9F	31	27	14	13	55.00	3.93	63.90	63.46	EBR 9M	24	13	12	52.00	4.00	60.80	60.67
EBR 10F	33	23	14	13	47.10	3.36	56.00	54.35	EBR 10M	26	17	16	44.80	2.64	53.60	50.40
EBR 11F	26	16	9	8	52.60	5.84	61.50	65.75	EBR 11M	19	13	12	57.30	4.41	66.10	66.85
EBR 12F	32	24	15	14	49.20	3.28	58.10	56.23	EBR 12M	20	12	11	56.60	4.72	65.40	66.89
EBR 13F	32	28	17	16	64.40	3.79	73.30	72.45	EBR 13M	16	7	6	16.70	2.39	25.50	22.27
–									EBR 13 + 2M	4	3	2	5.60	1.87	14.40	11.20
EBR 14F	44	28	17	16	52.60	3.09	61.50	59.18	EBR 14M	31	18	17	57.30	3.18	66.10	64.04
EBR 15F	31	24	16	15	56.60	3.54	65.50	64.15	EBR 15M	23	12	11	54.20	4.52	63.00	64.05
EBR 16F	28	21	13	12	48.00	3.69	56.90	56	EBR 16M	17	12	11	47.20	3.93	56.00	55.78
EBR 17F	40	28	15	14	58.60	3.91	67.50	66.97	EBR 17M	27	10	9	45.50	4.55	54.30	55.61
EBR 18F	35	20	14	13	59.00	4.21	67.90	68.08	EBR 18M	31	14	13	50.60	3.61	59.40	58.38
EBR 19F	31	20	14	13	44.70	3.19	53.60	51.58	EBR 19M	24	7	6	45.60	6.51	54.40	60.80
EBR 20F	25	20	13	12	47.20	3.63	56.10	55.07	EBR 20M	15	11	10	42.70	3.88	51.50	51.24
EBR 21F	25	15	10	9	42.80	4.28	51.70	52.31	EBR 21M	21	12	11	50.80	4.23	59.60	60.04
EBR 22F	25	15	8	7	51.70	6.46	60.60	66.47	EBR 22M	18	11	10	51.70	4.70	60.50	62.04
EBR 23F	32	26	16	15	51.60	3.23	60.50	58.48	EBR 23M	23	11	10	54.20	4.93	63.00	65.04
EBR 24F	10	3	3	2	11.20	3.73	20.10	22.4	EBR 24M	9	7	6	49.00	7.00	57.80	65.33
EBR 24 + 2F		2	2	1	1.10	0.55	10.00	3.3	–							
single marker	3	5								5						
Total	714	514	305	280	1249.8	103.28	1472.30	1479.59		517	285	259	1140.30	103.47	1369.10	1371.68
Average		21				4.1		1475.95		21				4.0		1370.39

Map distances are shown in centimorgans (cM)

*LG* linkage group, *G*_e1_ genome estimate size 1, *G*_e2_ genome estimate size 2

**Table 4:** Summary of the genetic linkage map of the kelp grouper

	Female	Male
Total number of markers scored	714	714
Number of markers mapped	509	512
Number of markers unmapped	5	5
Number of genetic linkages	24	24
Average number of markers per group	21	21
Minimum number of markers per group	5	9
Maximum number of markers per group	29	31
Minimum length of genetic linkage group (cM)	1.1	5.6
Maximum length of genetic linkage group (cM)	65.4	58
Observed genome length (cM)		
*G*_oa_	1249.8	1140.3
Average marker spacing (cM)	2.5	2.2
Average interval (cM)	4.1	4.0
Estimated genome length (cM)		
*G*_e1_	1472.30	1369.10
*G*_e2_	1479.59	1371.68
G _e_	1475.95	1370.39
Genome coverage %		
*C*_f_	84.68	83.21
Recombination rate	1.12	1

The recombination rate female/male (1.12:1)

*cM* centimorgan, *G*_oa_ observed genome length, *G*_e1_ genome estimate size 1, *G*_e2_ genome estimate size 2, *Ge* genome length estimate, *C*_*f*_ genome coverage of (%)

Recombination rate between the sex-specific genetic linkages were estimated by co-segregation markers. At least two SSR markers shared loci in the female and male maps and could be used to calculate the recombination rate among adjacently paired markers. The total length of genetic distance obtained from 24 genetic linkage groups (LGs) were 1249.8 and 1140.3 cM in female and male maps, respectively. The relative recombination ratio between females and males in these pairs was 1.12:1, which indicated that female LGs had a higher recombination rate than male except for LGs 1, 8, 11, 12, 14, and 19 (Table 5).

**Table 5:** Summary of genetic distances of co-segregation

List	LG	Common intervals	Genetic distance				
			Female^a^	Male^b^	F/M equivalent^c^	cM for female^d^	cM for male ^e^
1	*EBR1*	Ebr00236FRA/Ebr00386FRA	0	11.2	M	*54.9*	*58.0*
2		Ebr00386FRA/Ebr01245FRA	0	1.1	M		
3		Ebr01245FRA/Ebr00375FRA	14.6	19.9	M		
4		Ebr00375FRA/Ebr01148FRA	3.4	5.6	M		
5		Ebr01148FRA/Ebr00284FRA	6.7	3.4	F		
6		Ebr00284FRA/ElaSTR400DB	0	1.1	M		
7		ElaSTR400DB/Ebr00899FRA	9	7.9	F		
8		Ebr00899FRA/Ebr00190FRA	1.1	2.2	M		
9		Ebr00190FRA/EawSTR30DB	1.1	0	F		
10		EawSTR30DB/Ebr01085FRA	0	0	Equivalent		
11		Ebr01085FRA/Ebr01091FRA	0	0	Equivalent		
12		Ebr01091FRA/Ebr01062FRA	6.8	4.4	F		
13		Ebr01062FRA/EawSTR8DB	7.8	1.2	F		
14		EawSTR8DB/Ebr01263FRA	3.3	0	F		
15		Ebr01263FRA/EfuSTR309DB	1.1	0	F		
16		EfuSTR309DB/Ebr00063FRA	0	0	Equivalent		
17	EBR2	Bbr01006FRA/Ebr01281FRA	1.1	11.3	M	59.1	41.7
18		Ebr01281FRA/Ebr00185FRA	2.2	14.9	M		
19		Ebr00185FRA/Ebr00422FRA	3.4	1.1	F		
20		Ebr00422FRA/Ebr00257FRA	4.4	1.1	F		
21		Ebr00257FRA/Ebr01294FRA	6.7	2.2	F		
22		Ebr01294FRA/Ebr01128FRA	20.1	5.6	F		
23		Ebr01128FRA/Ebr01144FRA	5.6	3.3	F		
24		Ebr01144FRA/EguSTR129DB	5.6	1.1	F		
25		EguSTR129DB/Ebr00056FRA	10	1.1	F		
26		Ebr00056FRA/Ebr00069FRA	0	0	Equivalent		
27	EBR3	Ebr01315FRA/Ebr00384FRA	5.6	0	F	58.5	42.0
28		Ebr00384FRA/Ebr00293FRA	0	10.1	M		
29		Ebr00293FRA/Ebr01320FRA	2.3	3.5	M		
30		Ebr01320FRA/Ebr00114FRA	5.6	11.7	M		
31		Ebr00114FRA/Ebr00678FRA	0	0	Equivalent		
32		Ebr00678FRA/Ebr00005FRA	2.2	2.2	Equivalent		
33		Ebr00005FRA/Ebr00829FRA	17	6.7	F		
34		Ebr00829FRA/Ebr00320FRA	1.2	0	F		
35		Ebr00320FRA/EawSTR12DB	2.2	1.1	F		
36		EawSTR12DB/Ebr00116FRA	4.4	0	F		
37		Ebr00116FRA/Ebr00325FRA	2.3	0	F		
38		Ebr00325FRA/Ebr01405FRA	0	0	Equivalent		
39		Ebr01405FRA/EguSTR122_reDB	11.3	3.4	F		
40		EguSTR122_reDB/Ebr01056FRA	4.4	2.2			
41		Ebr01056FRA/Ebr01239FRA	0	1.1	M		
42	EBR4	Ebr00232FRA/Ebr00552FRA	0	0	Equivalent	61.3	47.3
43		Ebr00552FRA/Ebr00469FRA	0	4.6	M		
44		Ebr00469FRA/Ebr00751FRA	1.1	12.4	M		
45		Ebr00751FRA/Ebr00047FRA	10.1	11.3	M		
46		Ebr00047FRA/Ebr01021FRA	2.2	1.1	F		
47		Ebr01021FRA/Ebr01372FRA	0	0	Equivalent		
48		Ebr01372FRA/Ebr00812FRA	2.3	2.3	Equivalent		
49		Ebr00812FRA/Ebr00200FRA	19.9	7.8	F		
50		Ebr00200FRA/Ebr00052FRA	16.8	7.8	F		
51		Ebr00052FRA/Ebr00517FRA	2.2	0	F		
52		Ebr00517FRA/Ebr01019FRA	6.7	0	F		
53		Ebr01019FRA/Ebr00099FRA	0	0	Equivalent		
54		Ebr00099FRA/EawSTR58DB	0	0	Equivalent		
55		EawSTR58DB/EawSTR19DB	0	0	Equivalent		
56	EBR5	EawSTR20DB/Ebr01090FRA	2.2	0	F	24.8	14.5
57		Ebr01090FRA/Ebr00066FRA	18.1	4.4	F		
58		Ebr00066FRA/Ebr01288FRA	0	0	Equivalent		
59		Ebr01288FRA/Ebr00685FRA	3.4	3.4	Equivalent		
60		Ebr00345FRA/MmiSTR226DB	1.1	0	F		
61		MmiSTR226DB/Ebr00761FRA	0	0	Equivalent		
62		Ebr00761FRA/Ebr00776FRA	0	0	Equivalent		
63		Ebr00776FRA/Ebr00372FRA	0	1.1	M		
64		Ebr00372FRA/Ebr00474FRA	0	5.6	M		
65	EBR6	Ebr00980FRA/Ebr00203FRA	0	1.1	M	50.6	48.1
66		Ebr203FRA/Ebr00041FRA	0	0	Equivalent		
67		Ebr00041FRA/PmaSTR301DB	3.3	1.1	F		
68		PmaSTR301DB/ElaSTR392DB	9	1.1	F		
69		ElaSTR392DB/Ebr00287FRA	20.3	6.7	F		
70		Ebr00287FRA/EBR00734FRA	3.4	6.7	M		
71		Ebr00734FRA/Ebr00736FRA	1.1	0	F		
72		Ebr00736FRA/Ebr01187FRA	11.3	2.2	F		
73		Ebr01187FRA/Ebr01157FRA	2.2	19	M		
74		Ebr01157FRA/Ebr00282FRA	0	10.2	M		
75	EBR7	ElsSRT220DB/Ebr00850FRA	0	0	Equivalent	48.6	29.0
76		Ebr00850FRA/Ebr00149FRA	0	5.6	M		
77		Ebr00149FRA/Ebr00218FRA	0	0	Equivalent		
78		Ebr00218FRA/Ebr00158FRA	2.2	4.4	M		
79		Ebr00158FRA/EfuSTR319DB	0	2.3	M		
80		EfuSTR319DB/Ebr01022FRA	0	2.2	M		
81		Ebr01022FRA/Ebr001316FRA	16.1	4.5	F		
82		Ebr01316FRA/Ebr00693FRA	1.1	0	F		
83		Ebr00693FRA/Ebr00762FRA	11.3	4.4	F		
84		Ebr00762FRA/Ebr00352FRA	4.5	0	F		
85		Ebr00352FRA/Ebr00465FRA	11.2	3.4	F		
86		Ebr00465FRA/ElaSTR407DB	2.2	2.2	Equivalent		
87	*EBR8*	Ebr01362FRA/Ebr01086FRA	0	0	Equivalent	*43.0*	*51.0*
88		Ebr01086FRA/Ebr01201FRA	0	2.2	M		
89		Ebr01201FRA/Ebr00181FRA	4.5	14.9	M		
90		Ebr00181FRA/Ebr00204FRA	0	1.1	M		
91		Ebr00204FRA/Ebr00663FRA	12.4	16.1	M		
92		Ebr00663FRA/Ebr00963FRA	2.3	8.9	M		
93		Ebr00963FRA/Ebr00786FRA	1.1	1.1	Equivalent		
94		Ebr00786FRA/Ebr00797FRA	5.5	3.4	F		
95		Ebr00797FRA/EfuSTR328DB	17.2	3.3	F		
96	EBR9	EquSTR247DB/ElaSTR415DB	0	0	Equivalent	53.9	50.9
97		ElaSTR415DB/Ebr01370FRA	5.6	0	F		
98		Ebr01370FRA/Ebr00134FRA	4.5	0	F		
99		Ebr00134FRA/Ebr00199FRA	0	0	Equivalent		
100		Ebr00199FRA/Ebr00872FRA	0	0	Equivalent		
101		Ebr00872FRA/Ebr00764FRA	1.1	0	F		
102		Ebr00764FRA/Ebr01229FRA	0	1.1	M		
103		Ebr01229FRA/EquSTR148DB	4.5	4.5	Equivalent		
104		EquSTR148DB/Ebr00155FRA	0	0	Equivalent		
105		Ebr00155FRA/Ebr00807FRA	11.1	6.7	F		
106		Ebr00807FRA/EawSTR35DB	5.6	1.1	F		
107		EawSTR35DB/Ebr01400FRA	4.6	2.3	F		
108		Ebr01400FRA/EcoSTR231DB	8	5.7	F		
109		EcoSTR231DB/Ebr00531FRA	0	1.1	M		
110		Ebr00531FRA/EquSTR157DB	1.1	3.3	M		
111		EquSTR157DB/Ebr01290FRA	1.1	0	F		
112		Ebr01290FRA/Ebr00378FRA	6.7	16.1	M		
113		Ebr00378FRA/ElaSTR404DB	0	9	M		
114		ElaSTR404DB/Ebr00557FRA	0	0	Equivalent		
115	EBR10	Ebr00265FRA/Ebr00262FRA	0	4.5	M	43.7	43.7
116		Ebr00262FRA/Ebr00984FRA	2.2	11.3	M		
117		Ebr00984FRA/Ebr01032FRA	1.1	3.3	M		
118		Ebr01032FRA/Ebr00827FRA	2.3	3.3	M		
119		Ebr00827FRA/Ebr00629FRA	0	1.1	M		
120		Ebr00629FRA/EawSTR36DB	2.2	4.5	M		
121		EawSTR36DB/Ebr00974FRA	10.1	4.5	F		
122		Ebr00974FRA/Ebr00743FRA	3.4	0	F		
123		Ebr00743FRA/Ebr01013FRA	1.1	1.1	Equivalent		
124		Ebr01013FRA/Ebr00903FRA	0	0	Equivalent		
125		Ebr00903FRA/Ebr00317FRA	2.2	2.2	Equivalent		
126		Ebr00317FRA/Ebr01114FRA	8	5.7	F		
127		Ebr01114FRA/Ebr00636FRA	1.1	0	F		
128		Ebr00636FRA/EfuSRE339DB	5.6	1.1	F		
129		EfuSTR339DB/Ebr00774FRA	4.4	1.1	F		
130	*EBR11*	EawSTR49DB/Ebr00728FRA	0	0	Equivalent	*51.5*	*57.3*
131		Ebr00728FRA/Ebr00832FRA	0	0	Equivalent		
132		Ebr00832FRA/Ebr00267FRA	0	13.4	M		
133		Ebr00267FRA/Ebr00777FRA	6.7	28.2	M		
134		Ebr00777FRA/Ebr00982FRA	0	0	Equivalent		
135		Ebr00928FRA/Ebr01351FRA	18.6	10.1	F		
136		Ebr01351FRA/Ebr00687FRA	7.8	1.1	F		
137		Ebr00687FRA/Ebr01020FRA	18.4	4.5	F		
138	*EBR12*	Ebr00186FRA/Ebr00106FRA	0	0	Equivalent	*39.2*	*51.1*
139		Ebr00106FRA/Ebr01054FRA	3.4	26.2	M		
140		Ebr01054FRA/Ebr00573FRA	2.2	4.5	M		
141		Ebr00573FRA/Ebr00180FRA	0	0	Equivalent		
142		Ebr00180FRA/Ebr01027FRA	0	1.1	M		
143		Ebr01027FRA/Ebr00010FRA	15.7	13.7	F		
144		Ebr00010FRA/Ebr00992FRA	4.5	2.2	F		
145		Ebr00992FRA/Ebr00840FRA	0	1.1	M		
146		Ebr00840FRA/Ebr00179FRA	2.3	1.1	F		
147		Ebr00179FRA/Ebr01088FRA	4.4	0	F		
148		Ebr01088FRA/Ebr00793FRA	6.7	1.2	F		
149	EBR13	Ebr00292FRA/Ebr01380FRA	5.7	0	F	49.7	22.3
150		Ebr01380FRA/Ebr00826FRA	3.3	0	F		
151		Ebr00826FRA/Ebr01101FRA	10.3	1.1	F		
152		Ebr01101FRA/Ebr00575FRA	0	0	Equivalent		
153		Ebr00575FRA/Ebr01402FRA	4.5	0	F		
154		Ebr01402FRA/Ebr00263FRA	0	0	Equivalent		
155		Ebr00263FRA/EitSTR377DB	2.2	5.6	M		
156		EitSTR377DB/Ebr00500FRA	19.3	3.3	F		
157		Ebr00500FRA/ElaSTR225DB	2.2	4.5	M		
158		ElaSTR225DB/Ebr00861FRA	1.1	1.1	F		
159		Ebr00861FRA/Ebr00090FRA	0	1.1	M		
160		Ebr00090FRA/Ebr01190FRA	1.1	0	F		
161		Ebr00163FRA/Ebr00254FRA	0	4.5	M		
162		Ebr00254FRA/Ebr00971FRA	0	1.1	M		
163	*EBR14*	EfuSTR358DB/Ebr01107FRA	2.2	2.3	M	*35.9*	*54.0*
164		Ebr01107FRA/Ebr00783FRA	2.3	1.1	F		
165		Ebr00783FRA/Ebr01444FRA	3.3	9	M		
166		Ebr01444FRA/Ebr01174FRA	5.6	2.2	F		
167		Ebr01174FRA/Ebr01464FRA	1.1	0	F		
168		Ebr01464FRA/Ebr00235FRA	7.8	10.2	M		
169		Ebr00235FRA/Ebr00520FRA	2.3	4.4	M		
170		Ebr00520FRA/Ebr00303FRA	0	1.1	M		
171		Ebr00303FRA/Ebr00990FRA	2.2	5.7	M		
172		Ebr00990FRA/Ebr00209FRA	1.2	1.2	Equivalent		
173		Ebr00209FRA/Ebr01363FRA	3.3	2.2	F		
174		Ebr01363FRA/Ebr00187FRA	4.6	1.1	F		
175		Ebr00187FRA/Ebr00554FRA	0	12.4	M		
176		Ebr00554FRA/Ebr00024FRA	0	1.1	M		
177	EBR15	Ebr00380FRA/Ebr00819FRA	0	7.8	M	56.6	54.2
178		Ebr00819FRA/Ebr00222FRA	0	11.2	M		
179		Ebr00222FRA/Ebr01335FRA	0	0	Equivalent		
180		Ebr01335FRA/Ebr00008FRA	5.6	17.3	M		
181		Ebr00008FRA/Ebr00051FRA	3.3	4.5	M		
182		Ebr00051FRA/Ebr00504FRA	2.2	1.1	F		
183		Ebr00504FRA/Ebr00131FRA	1.2	1.1	F		
184		Ebr00131FRA/Ebr00072FRA	15.9	6.7	F		
185		Ebr00072FRA/Ebr01225FRA	8.1	3.4	F		
186		Ebr01225FRA/Ebr00244FRA	4.7	0	F		
187		Ebr00244FRA/Ebr01317FRA	0	0	Equivalent		
188		Ebr01317FRA/Ebr00064FRA	2.2	0	F		
189		Ebr00064FRA/Ebr00529FRA	1.1	0	F		
190		Ebr00529FRA/Ebr00876FRA	7.8	1.1	F		
191		Ebr00876FRA/Ebr00156FRA	4.5	0	F		
192	EBR16	Ebr00986FRA/Ebr00205FRA	3.3	2.2	F	48.0	46.1
193		Ebr00205FRA/EitSTR375DB	4.5	0	F		
194		EitSTR375FRA/Ebr00428FRA	0	0	Equivalent		
195		Ebr00428FRA/Ebr00138FRA	0	1.1	M		
196		Ebr00138FRA/EseSTR78DB	28	9	F		
197		EseSTR78DB/Ebr01421FRA	2.2	6.7	M		
198		Ebr01421FRA/Ebr01104FRA	2.2	4.4	M		
199		Ebr01104FRA/Ebr00939FRA	4.5	5.6	M		
200		Ebr00939FRA/EfuSTR360DB	3.3	17.1	M		
201	EBR17	ElaSTR411DB/Ebr00813FRA	0	0	Equivalent	58.6	45.5
202		Ebr00813FRA/Ebr00360FRA	0	1.1	M		
203		Ebr00360FRA/Ebr01210FRA	0	5.6	M		
204		Ebr01210FRA/EguSTR150DB	1.1	18.6	M		
205		EguSTR150DB/Ebr00896FRA	5.6	2.2	F		
206		Ebr00896FRA/EfuSTR420DB	5.6	0	F		
207		EfuSTR420DB/Ebr00153FRA	4.5	1.1	F		
208		Ebr00153FRA/Ebr00702FRA	4.5	0	F		
209		Ebr00702FRA/Ebr00314FRA	1.1	5.6	M		
210		Ebr00314FRA/EguSTR119DB	1.1	0	F		
211		EguSTR119DB/EcoSTR261DB	19.4	10.1	F		
212		EcoSTR261DB/Ebr00401FRA	0	0	Equivalent		
213		Ebr00401FRA/Ebr00012FRA	14.6	1.2	F		
214		Ebr00012FRA/EBR00207FRA	1.1	0	F		
215	EBR18	Ebr00202FRA/EitSTR378DB	0	0	Equivalent	59.0	47.2
216		EitSTR378DB/Ebr01340FRA	3.3	0	F		
217		Ebr01340FRA/Ebr00091FRA	0	0	Equivalent		
218		Ebr00091FRA/Ebr00241FRA	4.5	1.1	F		
219		Ebr00241FRA/Ebr00111FRA	11.3	3.3	F		
220		Ebr00111FRA/Ebr01356FRA	5.6	4.6	F		
221		Ebr01356FRA/ElaSTR405DB	1.1	0	F		
222		ElaSTR405DB/Ebr01212FRA	5.7	11.1	M		
223		Ebr01212FRA/Ebr00985FRA	0	0	Equivalent		
224		Ebr00985FRA/Ebr00443FRA	3.3	0	F		
225		Ebr00443FRA/Ebr00686FRA	5.6	1.1	F		
226		Ebr00686FRA/Ebr01005FRA	7	9	M		
227		Ebr01005FRA/Ebr01099FRA	11.6	0	F		
228		Ebr01099FRA/Ebr01336FRA	0	17	M		
229	**EBR19**	Ebr00855FRA/Ebr00724FRA	0	0	Equivalent	**44.7**	**45.6**
230		Ebr00724FRA/EquSTR126DB	0	0	Equivalent		
231		EquSTR126DB/Ebr00713FRA	0	9	M		
232		Ebr00713FRA/EacSTR234DB	4.5	19.9	M		
233		EacSTR234DB/PlaSTR269DB	3.3	1.1	F		
234		PlaSTR269DB/Ebr00508FRA	6.7	5.5	M		
235		Ebr00508FRA/Ebr00313FRA	0	0	F		
236		Ebr00313FRA/Ebr01172FRA	12.3	2.3	M		
237		Ebr01172FRA/Ebr01275FRA	13.5	7.8	M		
238		Ebr01275FRA/Ebr00533FRA	2.2	0	F		
239		Ebr00533FRA/Ebr00105FRA	1.1	0	F		
240		Ebr00105FRA/Ebr00333FRA	1.1	0	F		
241	EBR20	Ebr00912FRA/EfuSTR321DB	0	4.5	M	45.0	42.7
242		EfuSTR321DB/Ebr01309FRA	13.7	13.7	M		
243		Ebr01309FRA/Ebr00280FRA	6.8	6.7	F		
244		Ebr00280FRA/Ebr00269FRA	6.6	2.2	F		
245		Ebr00269FRA/Ebr00273FRA	0	0	Equivalent		
246		Ebr00273FRA/Ebr01024FRA	13.4	7.8	F		
247		Ebr01024FRA/EguSTR151DB	2.2	1.1	F		
248		EguSTR151DB/Ebr00043FRA	0	0	Equivalent		
249		Ebr00043FRA/Ebr00666FRA	2.3	6.7	M		
250	EBR21	Ebr01267FRA/EguSTR141DB	0	0	Equivalent	38.3	21.3
251		EguSTR141DB/Ebr00891FRA	0	1.2	M		
252		Ebr00891FRA/Ebr00192FRA	3.3	3.3	Equivalent		
253		Ebr00192FRA/Ebr00136FRA	0	0	Equivalent		
254		Ebr00136FRA/Ebr00212FRA	4.5	9	M		
255		Ebr00212FRA/Ebr01440FRA	13.7	3.3	F		
256		Ebr01440FRA/Ebr01255FRA	1.1	1.1	Equivalent		
257		Ebr01255FRA/Ebr00633FRA	0	0	Equivalent		
258		Ebr00633FRA/Ebr00067FRA	4.5	0	F		
259		Ebr00067FRA/Ebr00588FRA	11.2	3.4	F		
260	EBR22	Ebr00470FRA/EguSTR147DB	11.3	34.9	M	51.7	51.7
261		EguSTR147DB/ElaSTR398DB	10.1	11.3	M		
262		ElaSTR398DB/ElaSTR406DB	0	0	Equivalent		
263		ElaSTR406DB/Ebr00152FRA	4.5	3.3	F		
264		Ebr00152FRA/Ebr00558FRA	15.7	2.2	F		
265		Ebr00558FRA/Ebr00095FRA	7.9	0	F		
266		Ebr00095FRA/Ebr00868FRA	2.2	0	F		
267	EBR23	Ebr00045FRA/Ebr00249FRA	0	0	Equivalent	51.6	45.2
268		Ebr00249FRA/Ebr00461FRA	0	0	Equivalent		
269		Ebr00461FRA/Ebr01236FRA	0	3.3	M		
270		Ebr01236FRA/Ebr01118FRA	0	2.3	M		
271		Ebr01118FRA/Ebr00605FRA	0	1.1	M		
272		Ebr00605FRA/Ebr001176FRA	6.7	20.6	M		
273		Ebr01176FRA/Ebr00975FRA	1.1	0	F		
274		Ebr00975FRA/Ebr01008FRA	6.7	10.1	M		
275		Ebr01008FRA/Ebr01113FRA	1.2	0	F		
276		Ebr01113FRA/Ebr00926FRA	2.2	2.2	Equivalent		
277		Ebr00926FRA/ElaSTR413DB	1.1	0	F		
278		ElaSTR413DB/MmiSTR267DB	10.1	4.5	F		
279		MmiSTR267DB/Ebr01098FRA	11.3	0	F		
280		Ebr01098FRA/Ebr00538FRA	3.4	0	F		
281		Ebr00538FRA/Ebr01286FRA	5.5	0	F		
282		Ebr01286FRA/Ebr00817FRA	2.3	1.1	F		
283	EBR24	Ebr01003FRA/Ebr01361FRA	7.9	1.2	F	9.0	1.2
284		Ebr01366FRA/Ebr00758FRA	1.1	0	F		
		Total^f^				1137.2	1011.6
		Recombination ratio^g^				1.12	1

Map distances are shown in centimorgans (cM). Values in italics indicate the male linkage group had higher recombination rate than that of the female linkage group

^a^Genetic distance of co-segregation markers in female linkage group

^b^Genetic distance of co-segregation markers in male linkage group

^c^Which sex exhibits longer genetic distance between co-segregation markers

^d^Total length of common intervals in each female linkage group

^e^Total length of common intervals in each male linkage group

^f^Total length of common intervals in all 24 linkage groups

^g^Average ratio of recombination rate between females and males

Genome length (*G*_e_) was estimated as approximately 1475.95 and 1370.39 cM in the female and male maps, respectively. The female map was 1.07 times longer than the male map. Only nine LGs (1, 6, 11, 12, 14, 19, 21, 23, and 24) on the male map were longer than the female map. The genome coverages of the female and male maps were estimated at 84.68 and 83.21 %, respectively (Table 4).

### Screening Candidate QTL Regions

**Fig. 2:**
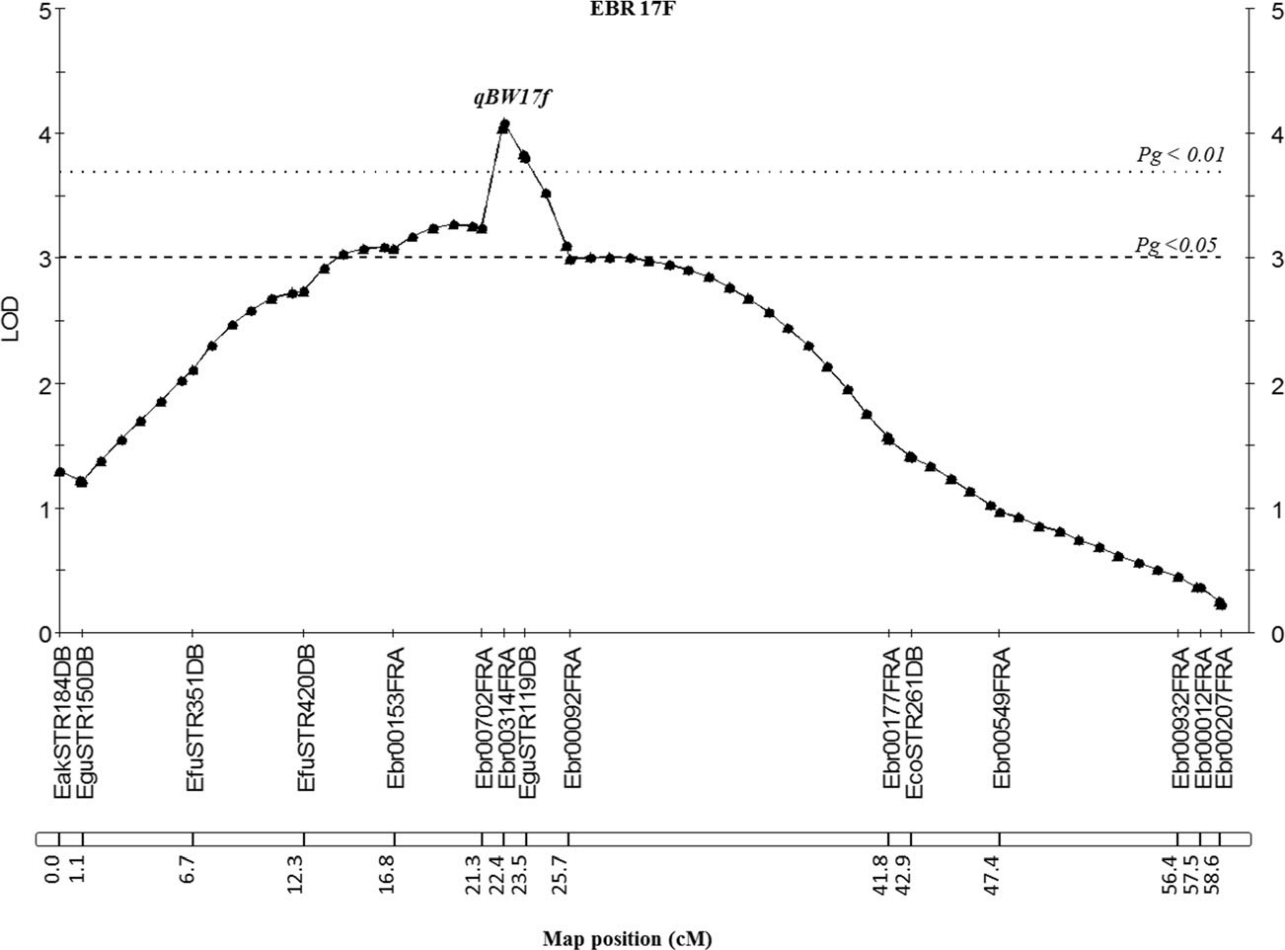
Localization of a significant marker for body weight traits in linkage group EBR 17F of family A. EBR (linkage group) F; marker distance on the female map. qBW17f: QTL for body weight on EBR 17F. Map positions and LOD scores were based on simple interval mapping. QTL analysis was performed using the software MapQTL 5. *LOD* limit of detection (significance threshold), *P*_*g*_ genome-wide significance threshold

**Fig. 3:**
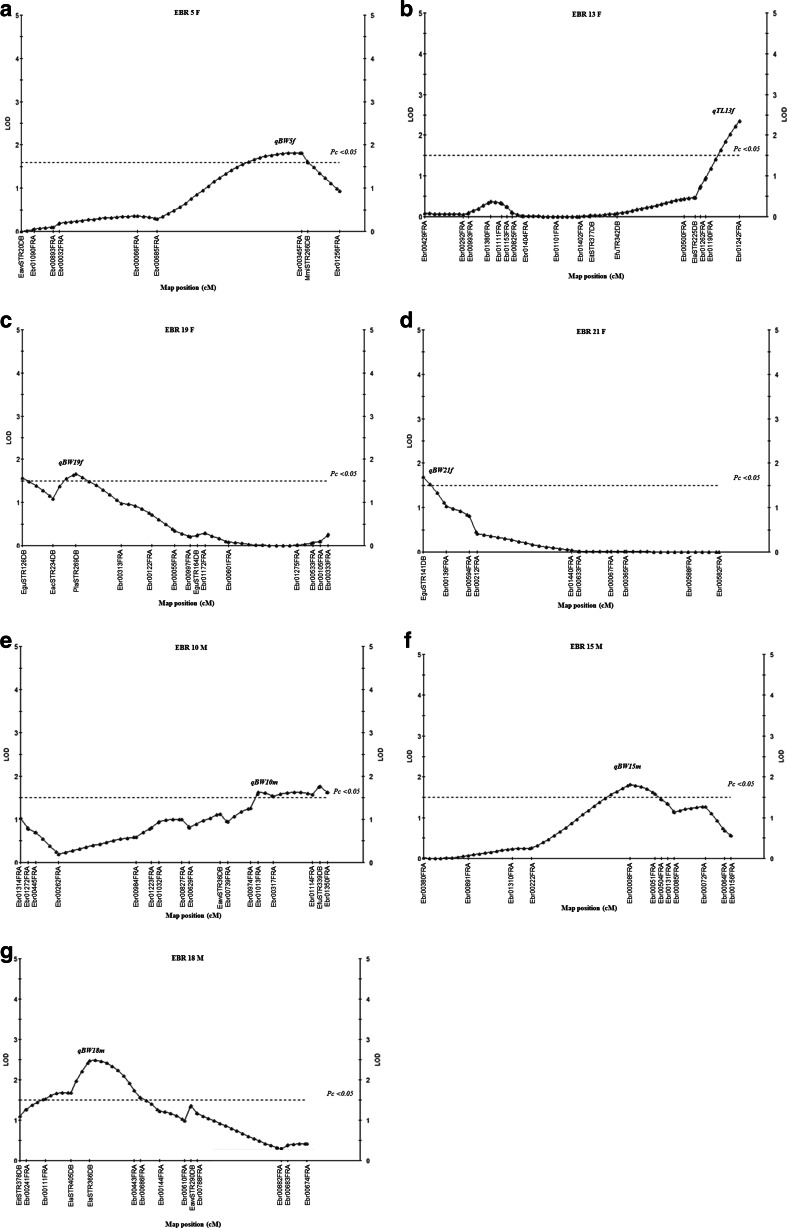
Localization of putative QTLs for body weight traits in the female map of family A. *EBR (linkage group) F* marker distance on the female map; *EBR (linkage group) M* marker distance on the male map. **a** qBW5f: QTL for body weight on EBR5F. **b** qBW13f: QTL for body weight on EBR13F. **c** qBW19f: QTL for body weight on EBR19F. **d** qBW21f: QTL for body weight on EBR 21F. **e** qBW10m: QTL for body weight on EBR 10M. **f** qBW15m: QTL for body weight on EBR 15M. **g** qBW18m: QTL for body weight on EBR 18M. Map positions and LOD scores were based on simple interval mapping, QTL analysis was performed using the software MapQTL 5. *LOD* limit of detection (significance threshold), *P*_*c*_ chromosome-wide significance threshold

Screening for candidate QTL of BW using the Kruskal-Wallis analysis of stage II family A (90 progeny) identified 5, 23, and 6 of the 34 total markers were significant (*P* < 0.01) on three linkage groups corresponding to chromosomes EBR 13F, EBR 17F, and EBR 18M (data not shown). The results of simple interval mapping and the permutation test showed a significant major QTL (qBW17f) at the 1 and 5 % genome-wide level on the linkage group EBR 17F (Fig. 2). The LOD score of qBW17f (LOD = 4.09) was higher than the genome-wide LOD significance threshold of 3.7. This candidate major QTL region was detected close to the SSR markers Ebr00153FRA, Ebr00702FRA, Ebr00314FRA, and EguSTR119DB, and accounted for 14.6–18.9 % of the phenotypic variance with 1.00–1.13 of the additive effect (Table 6). While seven putative QTLs (qBW5f, qBW10m, qBW13f, qBW15m, qBW18m, qBW19f, and qBW21f) were significant at a 5 % chromosome-wide level on linkage groups EBR 5F, EBR 13F, EBR 19F, and EBR 21F of the female map, and linkage groups EBR 10M, EBR 15M, and EBR 18M of the male map (Fig. 3) and could explain 7.5–12 % of the phenotypic variance with 0.70–0.92 of the additive effect (Table 6).

**Table 6:** Location of major and putative QTLs for body weight of the kelp grouper family A under genome-wide analysis

QTL	Sex	Trait	QTL name	LG	Locus name	LOD	LOD threshold		PVE (%)	Additive effect
							Genome-wide	Chromosome-wide		
Major	Female	Body weight	qBW17f	EBR 17F	Ebr00314FRA	4.09^b^	3.0 (3.7)	1.6	18.9	1.13
					EguSTR119DB	3.80^b^	3.0 (3.7)	1.6	17.7	1.10
					Ebr00702FRA	3.24^a^	3.0 (3.7)	1.6	15.2	1.01
					Ebr00153FRA	3.08^a^	3.0 (3.7)	1.6	14.6	1.00
Putative	Female	Body weight	qBW5f	EBR 5F	Ebr00345FRA	1.81^c^	3.0 (3.7)	1.6	8.9	0.76
					MimiSTR266DB	1.60^c^	3.0 (3.7)	1.6	7.8	0.71
					Ebr00253FRA	1.60^c^	3.0 (3.7)	1.6	7.8	0.71
					Ebr00270FRA	1.60^c^	3.0 (3.7)	1.6	7.8	0.71
					Ebr00305FRA	1.60^c^	3.0 (3.7)	1.6	7.8	0.71
					Ebr00372FRA	1.60^c^	3.0 (3.7)	1.6	7.8	0.71
					Ebr00474FRA	1.60^c^	3.0 (3.7)	1.6	7.8	0.71
					Ebr00761FRA	1.60^c^	3.0 (3.7)	1.6	7.8	0.71
					Ebr00776FRA	1.60^c^	3.0 (3.7)	1.6	7.8	0.71
			qBW13f	EBR 13F	Ebr01242FRA	2.50^c^	3.0 (3.7)	1.6	12	0.92
					Ebr00971FRA	2.50^c^	3.0 (3.7)	1.6	12	0.92
					Ebr00254FRA	2.50^c^	3.0 (3.7)	1.6	12	0.92
					Ebr00163FRA	2.50^c^	3.0 (3.7)	1.6	12	0.92
					Ebr00147FRA	2.50^c^	3.0 (3.7)	1.6	12	0.92
			qBW19f	EBR 19F	PlaSTR269DB	1.66^c^	3.0 (3.7)	1.5	8.1	0.74
			qBW21f	EBR 21F	EquSTR141DB	1.69^c^	3.0 (3.7)	1.5	8.3	0.73
					Ebr00891FRA	1.69^c^	3.0 (3.7)	1.5	8.3	0.73
					Ebr00924FRA	1.69^c^	3.0 (3.7)	1.5	8.3	0.73
					Ebr01267FRA	1.69^c^	3.0 (3.7)	1.5	8.3	0.73
	Male	Body weight	qBW10m	EBR 10M	Ebr01013FRA	1.63^c^	3.0 (3.7)	1.5	8	0.72
					Ebr00903FRA	1.63^c^	3.0 (3.7)	1.5	8	0.72
					Ebr00317FRA	1.53^c^	3.0 (3.7)	1.5	7.6	0.70
					Ebr01114FRA	1.57^c^	3.0 (3.7)	1.5	7.7	0.71
					Ebr00712FRA	1.57^c^	3.0 (3.7)	1.5	7.7	0.71
					Ebr00636FRA	1.57^c^	3.0 (3.7)	1.5	7.7	0.71
					EfuSTR339DB	1.76^c^	3.0 (3.7)	1.5	8.6	0.78
					Ebr01350FRA	1.62^c^	3.0 (3.7)	1.5	7.9	0.72
					Ebr00774FRA	1.62^c^	3.0 (3.7)	1.5	7.9	0.72
			qBW15m	EBR 15M	Ebr00008FRA	1.81^c^	3.0 (3.7)	1.5	8.8	0.76
					Ebr00051FRA	1.58^c^	3.0 (3.7)	1.5	7.7	0.72
			qBW18m	EBR 18M	Ebr00111FRA	1.53^c^	3.0 (3.7)	1.5	7.5	0.71
					ElaSTR405DB	1.68^c^	3.0 (3.7)	1.5	8.2	0.74
					Ebr00414FRA	1.68^c^	3.0 (3.7)	1.5	8.2	0.74
					Ebr00698FRA	1.68^c^	3.0 (3.7)	1.5	8.2	0.74
					Ebr01208FRA	1.68^c^	3.0 (3.7)	1.5	8.2	0.74
					Ebr01356FRA	1.68^c^	3.0 (3.7)	1.5	8.2	0.74
					ElaSTR366DB	2.47^c^	3.0 (3.7)	1.5	11.9	0.88
					Ebr00443FRA	1.74^c^	3.0 (3.7)	1.5	8.5	0.74
					Ebr00985FRA	1.74^c^	3.0 (3.7)	1.5	8.5	0.74
					Ebr01212FRA	1.74^c^	3.0 (3.7)	1.5	8.5	0.74
					Ebr00686FRA	1.55^c^	3.0 (3.7)	1.5	7.6	0.70
					Ebr00944FRA	1.55^c^	3.0 (3.7)	1.5	7.6	0.70

*Signif* significance levels. *PVE (%)* the percentage of the variance explained by QTL

^a^Experiment-wide significant QTL (*P* < 0.05)

^b^Experiment-wide significant QTL (*P* < 0.01)

^c^Chromosome-wide significant QTL (*P* < 0.05)

Due to a high significance level (*P* < 0.01) in the non-parametric K-W analysis and the LOD score of a candidate major and two putative QTLs exceeded the high significance thresholds (*P* < 0.01 and *P* < 0.05) of genome- and chromosome-wide after simple interval mapping and permutation analysis. Then, 35 representative microsatellite markers (Additional file 3) related with these QTLs from three linkage groups (EBR 13F , EBR 17F, and EBR 18M) were used to confirm the candidate QTL regions in all progeny in the two stages of families A and B.

### Confirmation of the Candidate QTL Regions

**Fig. 4:**
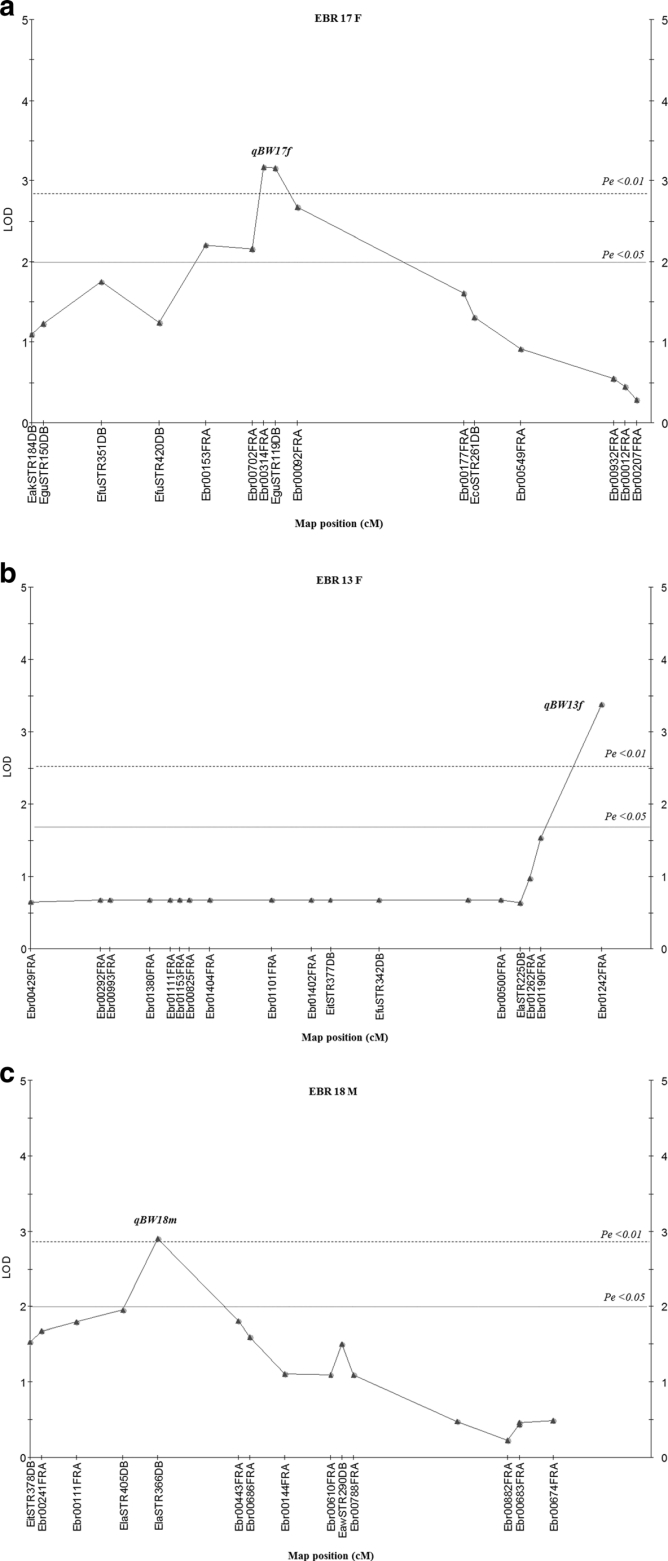
Localization of major and putative QTLs for the body weight trait in the female and male maps, based on confirmed QTL regions of family A. *EBR (linkage group) F* marker distance on the female map; *EBR (linkage group) M* marker distance on the male map. **a** qBW17f: QTL for body weight on EBR17F. **b** qBW13f: QTL for body weight on EBR 13F. **c** qBW18m: QTL for body weight on EBR 18M. Map positions and LOD scores were based on a simple interval mapping. QTL analysis was performed using the software MapQTL 5. *LOD* limit of detection (significance threshold), *P*_e_ experiment-wide significance threshold, *P*_*c*_ chromosome-wide significance threshold

Thirty-five marker loci from three candidate QTL regions of three linkage groups affecting BW in stage II family A were used to confirm the QTL region in the other stage of the same family and in the other family by collecting genotype data in both stages of the two families. In the case of the stage II family analysis, the number of progeny analyzed for the trait analysis increased from 90 to 163 progeny. For family A, the K-W test results showed that eight markers from linkage groups EBR 13F and EBR 17F of the female map showed consistently significant results (*P* < 0.001) in stage II. Of them, three markers (Ebr00254FRA, Ebr00314FRA, and EguSTR119DB) showed the highest consistently significant results (*P* < 0.0005), while only two markers (ElaSTR366DB and Ebr00443FRA) showed consistently significant results (*P* < 0.005) in the male map (Table 7). Simple interval mapping on a chromosome-wide basis was then performed in each stage. The results showed only three QTLs (qBW13f, qBW17f, and qBW18m) in stage II were still significant. However, the results of interval mapping in stage II showed decreasing LOD scores (4.09 to 3.17) from the genome-wide analysis, with an LOD experimental-wide significance threshold of 2.0 (Fig. 4a) with the LOD maximum locus (qBW17f) could explain phenotypic variance ranging 5.9–8.6 % with 0.49–0.59 of the additive effect of the BW traits. In contrast, for two candidate QTLs (qBW13f, qBW18m) on linkage groups EBR 13F and EBR 18M, their LOD scores increased from 2.5 to 3.38, and from 2.47 to 2.9, respectively, under the experiment-wide analysis. LOD significant threshold of 2.0 and 2.0 (Fig. 4b, c) with the region of LOD maximum locus (qBW13f and qBW18m) could explain phenotypic variance ranging 4.2–9.1 and 5–7.9 % with 0.42–0.62 and 0.44–0.56 of the additive effect of BW traits (Table 8). Nevertheless, we could not find any consistently significant results in stage I of family A.

**Table 7:** Significant markers for body weight in stage I and II of families A and B using Kruskal-Wallis analysis

Linkage group	Position	Locus	Candidate QTL region		Stage I family A female		Stage I family A male		Stage II family A female		Stage II family A male		Stage I family B female		Stage I family B male		Stage II family B female		Stage II family B male	
			K*	Signif.	K*	Signif.	K*	Signif.	K*	Signif.	K*	Signif.	K*	Signif.	K*	Signif.	K*	Signif.	K*	Signif.
EBR 13F	64.387	Ebr00254FRA	8.96	****	0.055	NS	0.042	NS	14.949	******	1.344	NS	–	–	0.048	NS	–	–	–	–
EBR 17F	0	Ebr01210FRA	5.883	**	0.059	NS	0.065	NS	4.489	**	1.241	NS	0.045	NS	0.336	NS	0.034	NS	2.387	NS
	1.111	EguSTR150DB	5.841	**	1.511	NS	0.172	NS	4.815	**	0.88	NS	1.503	NS	4.788	**	0.005	NS	0.025	NS
	6.69	Ebr00896FRA	10.16	****	1.086	NS	0.193	NS	8.386	****	0.232	NS	0.069	NS	0.773	NS	0.008	NS	1.304	NS
	12.332	EfuSTR420DB	11.696	*****	0.063	NS	0.339	NS	7.375	***	0.013	NS	1.016	NS	4.561	**	0.867	NS	0.002	NS
	16.838	Ebr00153FRA	14.717	******	0.181	NS	0.557	NS	9.307	****	0.083	NS	1.121	NS	6.96	***	0.005	NS	0.466	NS
	21.294	Ebr00702FRA	14.322	******	0.313	NS	0.829	NS	9.558	****	0.027	NS	3.186	NS	11.117	*****	0.541	NS	0.117	NS
	22.406	Ebr00314FRA	17.3	*******	0.283	NS	0.546	NS	13.273	******	0.095	NS	2.764	NS	4.377	**	0.116	NS	0.404	NS
	23.517	EguSTR119DB	16.081	*******	0.481	NS	0.554	NS	13.125	******	0.067	NS	3.549	NS	5.725	**	0.116	NS	0.404	NS
	25.741	Ebr00092FRA	12.637	******	0.675	NS	–	–	10.891	*****	–	–	3.198	NS	–	–	0.828	NS	–	–
	41.83	Ebr00177FRA	7.61	***	0.071	NS	–	–	9.302	****	–	–	–	–	–	–	–	–	–	–
	42.941	EcoSTR261DB	6.836	***	0.391	NS	1.851	NS	7.521	***	0.002	NS	2.516	NS	1.919	NS	1.503	NS	1.795	NS
	47.397	Ebr00549FRA	4.857	**	0.394	NS	–	–	5.136	**	–	–	5.546	**	–	–	0.909	NS	–	–
	57.493	Ebr00012FRA	2.098	NS	0.235	NS	1.946	NS	2.619	NS	0.164	NS	0.564	NS	5.191	**	1.139	NS	0.227	NS
	58.604	Ebr00207FRA	1.37	NS	0.597	NS	1.521	NS	1.676	NS	0.164	NS	–	–	3.97	**	–	–	0.227	NS
	0	Ebr00091FRA	4.177	**	0.472	NS	3.059	NS	1.223	NS	6.377	**	0.65	NS	0.47	NS	1.681	NS	0.076	NS
EBR 18M	1.111	Ebr00241FRA	4.847	**	1.88	NS	3.762	NS	0.551	NS	7.085	***	1.404	NS	0.47	NS	3.74	NS	0.076	NS
	4.45	Ebr00111FRA	6.297	**	0.526	NS	3.605	NS	0.082	NS	7.646	***	0.771	NS	0.249	NS	5.248	**	0.096	NS
	8.956	ElaSTR405DB	6.915	***	0.403	NS	2.049	NS	0.062	NS	7.714	***	1.282	NS	0.533	NS	5.337	**	0.294	NS
	12.294	ElaSTR366DB	9.977	****	–	–	4.075	**	–	–	12.209	******	3.346	NS	0.531	NS	–	–	0.119	NS
	20.136	Ebr00443FRA	8.276	****	0.015	NS	5.562	**	0.421	NS	8.648	****	1.8	NS	0.112	NS	4.5	**	0.002	NS
	21.247	Ebr00686FRA	7.356	***	0.118	NS	4.38	**	2.038	NS	7.48	***	–	–	0.042	NS	–	–	0.001	NS
	24.585	Ebr00144FRA	6.026	**	–	–	4.63	**	–	–	5.38	**	–	–	0.104	NS	–	–	0.001	NS
	29.042	Ebr00610FRA	5.014	**	–	–	3.767	NS	–	–	4.857	**	–	–	0.003	NS	–	–	0.26	NS
	30.153	Ebr01099FRA	6.647	***	0.154	NS	3.549	NS	2.265	NS	6.458	**	–	–	–	–	–	–	–	–
	31.264	Ebr00788FRA	5.537	**	–	–	2.133	NS	–	–	4.215	**	–	–	1.006	NS	–	–	0.101	NS
	47.243	Ebr01276FRA	1.465	NS	–	–	3.864	**	–	–	1.566	NS	–	–	0.698	NS	–	–	0.301	NS

*Signif* significance levels, K* Kruskal-Wallis test statistic K*, *NS* not significant, – no polymorphism in this marker, *EBR(linkage group)F* dam allele in female linkage group, *EBR(linkage group)M* sire allele in male linkage group

**<0.05

***<0.01

****<0.005

*****<0.001

******<0.0005

*******<0.0001

**Table 8:** Location of major and putative QTLs in the linkage map of the kelp grouper under experiment-wide analysis

Trait	QTL	Family	Stage	Sex	QTL name	LG	Locus name	LOD	LOD threshold		PVE (%)	Additive effect
									Experiment-wide	Chromosome-wide		
Body weight	Major	A	II	Female	qBW17f	EBR 17F	Ebr00314FRA	3.17^b^	2.0(2.8)	1.7	8.6	0.59
							EguSTR119DB	3.16^b^	2.0(2.8)	1.7	8.5	0.59
							Ebr00702FRA	2.16^a^	2.0(2.8)	1.7	5.9	0.49
							Ebr00153FRA	2.21^a^	2.0(2.8)	1.7	6	0.49
	Putative	B	I	Male	qBW17m-1	EBR 17M	Ebr00153FRA	1.69	3.0(3.8)	2.0	6.7	0.52
							Ebr00702FRA	2.65^a^	3.0(3.8)	2.0	10.3	0.64
							EquSTR119DB	1.33	3.0(3.8)	2.0	5.3	0.46
	Putative	A	II	Female	qBW13f	EBR 13F	Ebr00500FRA	0.67	2.0(2.8)	1.3	1.9	0.28
							EguSTR225DB	0.63	2.0(2.8)	1.3	1.8	0.26
							Ebr00861FRA	0.98	2.0(2.8)	1.3	2.7	0.33
							Ebr1190FRA	1.53^c^	2.0(2.8)	1.3	4.2	0.42
							Ebr00254FRA	3.38^b^	2.0(2.8)	1.3	9.1	0.62
		A	II	Male	qBW18m	EBR 18M	ElaSTR405DB	1.95^c^	2.0(2.8)	1.6	5.4	0.47
							ElaSTR366DB	2.9^b^	2.0(2.8)	1.6	7.9	0.56
							Ebr00443FRA	1.81^c^	2.0(2.8)	1.6	5	0.44
Total length	Major	A	II	Female	qTL17f	EBR 17F	Ebr00314FRA	3.25^b^	2.0(2.8)	1.6	8.8	0.59
							EguSTR119DB	3.18^b^	2.0(2.8)	1.6	8.6	0.59
							Ebr00153FRA	2.52^a^	2.0(2.8)	1.6	6.9	0.53
							Ebr00702FRA	2.24^a^	2.0(2.8)	1.6	6.1	0.50
							Ebr00092FRA	2.92^b^	2.0(2.8)	1.6	7.9	0.57
	Putative	A	II	Female	qTL13f	EBR 13F	Ebr00500FRA	0.46	2.0(2.8)	1.2	1.3	0.23
							EguSTR225DB	0.44	2.0(2.8)	1.2	1.2	0.22
							Ebr00861FRA	0.79	2.0(2.8)	1.2	2.2	0.30
							Ebr1190FRA	1.39^c^	2.0(2.8)	1.2	3.9	0.40
							Ebr00254FRA	3.24^b^	2.0(2.8)	1.2	8.7	0.61
	Putative	A	II	Male	qTL18m	EBR 18M	ElaSTR405DB	1.41	2.0(2.8)	1.5	3.9	0.40
							ElaSTR366DB	2.46^a^	2.0(2.8)	1.5	6.7	0.52
							Ebr00443FRA	1.73^c^	2.0(2.8)	1.5	4.8	0.43

*Signif* significance levels; *PVE (%)* the percentage of the variance explained by QTL

^a^Experiment-wide significant QTL (*P* < 0.05)

^b^Experiment-wide significant QTL (*P* < 0.01)

^c^Chromosome-wide significant QTL (*P* < 0.05)

**Fig 5:**
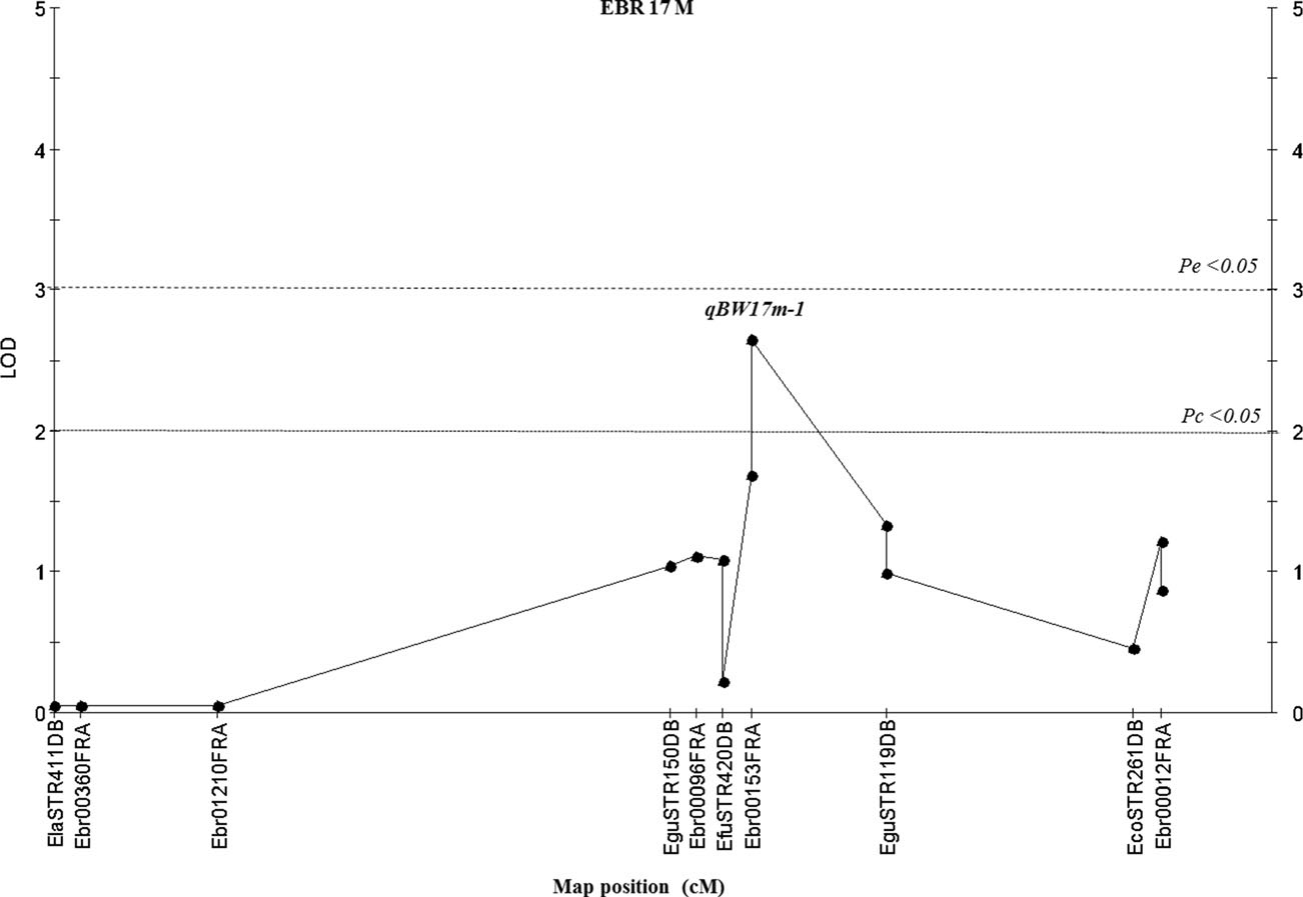
Localization of a suggested QTL for body weight traits in the male map of family B. *EBR (linkage group) M* marker distance on the male map. qBW17m-1: QTL number 1 for body weight on EBR 17M; Map positions and LOD score based on simple interval mapping. QTL analysis was performed using the software MapQTL 5. *LOD* limit of detection (significance threshold), *P*_*e*_ experiment-wide significance threshold, *P*_*c*_; chromosome-wide significance threshold

In family B, the results showed only one marker (Ebr00702FRA) on linkage group EBR 17M in stage I, which presented consistent highly significant results (*P* < 0.001), was a putative QTL (qBW17m-1). It had a LOD score of 2.65, which was higher than the chromosome-wide LOD significance threshold of 2.0, with a range of 10.3 % of the phenotypic variance with 0.64 of the additive effect (Table 8, Fig. 5). Meanwhile, other significant regions in all linkages were rejected as QTLs in stage II of family B.

### Association of Growth-Related Trait QTL Regions and TL

In this study, we also measured another phenotype, TL, which was highly correlated with BW of fish (Pearson correlation coefficient test *P* < 0.01), particularly in stage II of both families. For stage II of family A, the results of the K-W analysis and simple interval mapping showed significant loci in eight linkage groups (EBR 5F, EBR 7F, EBR 8F, EBR 13F, EBR 10M, EBR 17F, EBR 18M, and EBR 22M). The LOD score of a major QTL (qTL17f) effected to TL in linkage group EBR 17F was 4.0. with genome-wide significance (*P* < 0.01). This QTL region could explain 14.7–18.5 % of the phenotypic variance and 0.99–1.12 of the additive effect of TL trait. Meanwhile, another region with an LOD maximum locus in the other linkage group had a value that exceeded the chromosome-wide value and could explain 7.0–11.3 % of the phenotypic variance and 0.69–0.89 of the additive effect of the TL trait (Table 9). Moreover, we confirmed all the candidate QTL regions that affected TL using 35 markers in both stages of families A and B, just as we did for the BW trait. The K-W analysis results revealed eight markers from linkage groups EBR 13F and EBR 17F of the female map that showed consistently significant results in stage II. Of them, three markers (Ebr00254FRA, Ebr00314FRA, and EguSTR119DB) showed the highest consistently significant results (*P* < 0.0005). While only two markers (ElaSTR366DB and Ebr00443FRA) in linkage group EBR 18M of the male map showed consistently significant results (*P* < 0.005) (Table 10). LOD analysis showed a decreasing LOD score from 4.00 to 3.25 at an LOD experimental-wide significance threshold of 2.0, in the candidate major QTL (qBW17f) on the linkage group EBR 17F. By contrast, the confirmation of two candidate putative QTL regions (qTL13f and qTL18m) demonstrated LOD scores that increased from 2.34 to 3.24 and 2.32 to 2.46, respectively, on the experiment-wide scale. LOD significant threshold of 2.0 and 2.0. The region of the LOD maximum locus (qTL13f and qTL18m) could explain phenotypic variance ranging from 3.9–8 .to 4.8–6.7 % of the phenotypic variance and 0.40–0.61 and 0.43–0.52 of the additive effect of the TL trait. As with the results for BW, we could not find any consistently significant values for stage I of family A or for both stages of family B (Table 8).

**Table 9:** Location of major and putative QTLs for total length of the kelp grouper family A under genome-wide analysis

QTL	Sex	Trait	QTL name	LG	Locus name	LOD	LOD threshold		PVE (%)	Additive effect
							Genome-wide	Chromosome-wide		
Major	Female	Total length	qTL17f	EBR 17F	Ebr00314FRA	4.00^b^	3.0 (4.0)	1.6	18.5	1.12
					EguSTR119DB	3.72^a^	3.0 (4.0)	1.6	17.3	1.09
					Ebr00153FRA	3.29^a^	3.0 (4.0)	1.6	15.5	1.03
					Ebr00702FRA	3.20^a^	3.0 (4.0)	1.6	15.1	1.00
					Ebr00092FRA	3.10^a^	3.0 (4.0)	1.6	14.7	0.99
Putative	Female	Total length	qTL5f	EBR 5F	Ebr000345FRA	1.42^c^	3.0 (4.0)	1.4	7	0.77
			qTL7f	EBR 7F	Ebr00352FRA	1.54^c^	3.0 (4.0)	1.5	7.6	0.70
					Ebr01043FRA	1.50^c^	3.0 (4.0)	1.5	7.7	0.69
			qTL8f	EBR 8F	Ebr00181FRA	1.56^c^	3.0 (4.0)	1.5	7.7	0.71
					Ebr00204FRA	1.56^c^	3.0 (4.0)	1.5	7.7	0.71
			qTL13f	EBR 13F	Ebr01242FRA	2.34^c^	3.0 (4.0)	1.5	11.3	0.89
					Ebr00971FRA	2.34^c^	3.0 (4.0)	1.5	11.3	0.89
					Ebr00254FRA	2.34^c^	3.0 (4.0)	1.5	11.3	0.89
					Ebr00163FRA	2.34^c^	3.0 (4.0)	1.5	11.3	0.89
					Ebr00147FRA	2.34^c^	3.0 (4.0)	1.5	11.3	0.89
	Male	Total length	qTL10m	EBR 10M	Ebr01013FRA	1.54^c^	3.0 (4.0)	1.5	7.6	0.70
					Ebr00903FRA	1.54^c^	3.0 (4.0)	1.5	7.6	0.70
			qTL18m	EBR 18M	ELaSTR366DB	2.32^c^	3.0 (4.0)	1.5	11.2	0.86
					Ebr00443FRA	1.80^c^	3.0 (4.0)	1.5	8.8	0.75
					Ebr00985FRA	1.80^c^	3.0 (4.0)	1.5	8.8	0.75
					Ebr01212FRA	1.80^c^	3.0 (4.0)	1.5	8.8	0.75
					Ebr00686FRA	1.59^c^	3.0 (4.0)	1.5	7.8	0.71
					Ebr00944FRA	1.59^c^	3.0 (4.0)	1.5	7.8	0.71
			qTL22m	EBR 22M	Ebr00622FRA	1.57^c^	3.0 (4.0)	1.5	7.7	0.71
					Ebr00773FRA	1.52^c^	3.0 (4.0)	1.5	7.5	0.70

*Signif* significance levels; *PVE* (%) the percentage of the variance explained by QTL

^a^Genome-wide significant QTL (*P* < 0.05)

^b^Genome-wide significant QTL (*P* < 0.01)

^c^Chromosome-wide significant QTL (*P* < 0.05)

**Table 10:** Significant markers for total length for stages I and II of families A and B using Kruskal-Wallis analysis

Linkage group	Position	Locus	Candidate QTL region		Stage I family A female		Stage I family A male		Stage II family A female		Stage II family A male		Stage I family B female		Stage I family B male		Stage II family B female		Stage II family B male	
			K*	Signif.	K*	Signif.	K*	Signif.	K*	Signif.	K*	Signif.	K*	Signif.	K*	Signif.	K*	Signif.	K*	Signif.
EBR 13F	57.526	Ebr01190FRA	4.013	**	0.123	NS	0.61	NS	6.52	**	1.209	NS	0.175	NS	0.838	NS	0.094	NS	0.676	NS
	64.387	Ebr00254FRA	10.009	****	0.249	NS	0.225	NS	14.501	******	1.437	NS	–	–	1.181	NS	–	–	–	–
EBR 17F	0	Ebr01210FRA	8.662	****	2.937	NS	0	NS	6.283	**	0.311	NS	0.003	NS	0	NS	0.138	NS	3.92	**
	1.111	EguSTR150DB	8.051	****	6.432	**	0.185	NS	6.718	***	0.393	NS	0.735	NS	0.796	NS	0.218	NS	0.087	NS
	6.69	Ebr00896FRA	10.819	****	4.698	**	0.021	NS	8.711	****	0.177	NS	0.196	NS	0.026	NS	0.311	NS	1.359	NS
	12.332	EfuSTR420DB	12.372	******	2.489	NS	0.064	NS	7.78	***	0.115	NS	0.441	NS	0.932	NS	1.265	NS	0.133	NS
	16.838	Ebr00153FRA	15.485	*******	4.397	**	0.515	NS	9.956	****	0.003	NS	0.324	NS	1.717	NS	0.047	NS	0.83	NS
	21.294	Ebr00702FRA	14.756	******	5.314	**	1.541	NS	9.583	****	0.003	NS	1.576	NS	2.52	NS	0.955	NS	0.033	NS
	22.406	Ebr00314FRA	17.642	*******	2.814	NS	0.623	NS	13.342	******	0.204	NS	4.394	**	0.615	NS	0.002	NS	0.633	NS
	23.517	EguSTR119DB	16.378	*******	4.249	**	1.285	NS	13.22	******	0.229	NS	3.657	NS	2.021	NS	0.002	NS	0.633	NS
	25.741	Ebr00092FRA	13.978	******	2.372	NS	–	–	11.673	*****	–	–	4.015	**	–	–	0.392	NS	–	–
	41.83	Ebr00177FRA	8.288	****	0.33	NS	–	–	11.045	*****	–	–	–	–	–	–	–	–	–	–
	42.941	EcoSTR261DB	7.107	***	0.099	NS	2.232	NS	9.184	****	0.004	NS	4.882	**	0.212	NS	0.889	NS	1.798	NS
	47.397	Ebr00549FRA	3.982	**	0.466	NS	–	–	6.356	**	–	–	7.98	****	–	–	0.58	NS	–	–
	56.381	Ebr00932FRA	1.298	NS	0.052	NS	–	–	3.491	NS	–	–	6.38	**	–	–	2.327	NS	–	–
	58.604	Ebr00207FRA	0.627	NS	0.253	NS	4.287	**	1.892	NS	0.091	NS	–	–	0.758	NS	–	–	0.207	NS
EBR 18M	1.111	Ebr00241FRA	4.162	**	0.142	NS	1.51	NS	0.542	NS	5.027	**	0.615	NS	1.181	NS	3.658	NS	0.058	NS
	4.45	Ebr00111FRA	4.785	**	0.756	NS	0.758	NS	0.365	NS	5.162	**	0.143	NS	0.732	NS	5.53	**	0.082	NS
	8.956	ElaSTR405DB	5.39	**	0.493	NS	0.47	NS	0	NS	5.584	**	0.121	NS	1.874	NS	5.907	**	0.21	NS
	12.294	ElaSTR366DB	9.191	****	–	–	1.745	NS	1.175	NS	10.067	****	–	–	1.184	NS	–	–	0.344	NS
	20.136	Ebr00443FRA	8.245	****	0.027	NS	2.666	NS	0.067	NS	7.953	****	0.816	NS	0.388	NS	4.507	**	0.002	NS
	21.247	Ebr00686FRA	7.195	***	0.459	NS	2.171	NS	0.781	NS	6.939	***	–	–	0.225	NS	–	–	0.005	NS
	24.585	Ebr00144FRA	5.36	**	-	-	2.692	NS	NS	NS	4.68	**	-	-	0.252	NS	-	-	0.005	NS
	29.042	Ebr00610FRA	4.914	**	–	–	1.568	NS	NS	NS	4.626	**	–	–	0.59	NS	–	–	0.124	NS
	30.153	Ebr01099FRA	6.492	**	0.202	NS	2.67	NS	1.032	NS	6.519	**	–	–	–	–	–	–	–	–
	31.264	Ebr00788FRA	5.395	**	–	–	1.019	NS	NS	NS	5.081	**	–	–	5.363	**	–	–	0.159	NS

S*ignif* significance levels, K* Kruskal-Wallis test statistic K*, *NS* not significant, – no polymorphism in this marker, *EBR(linkage group) F* dam allele in female linkage group, *EBR(Linkage group) M* M is sire allele in male linkage group

**<0.05

***<0.01

****<0.005

*****<0.001

******<0.0005

*******<0.0001

## Discussion

The high-resolution genetic linkage maps of the kelp grouper produced in this study greatly enhanced the previous genetic linkage map for the kelp grouper which was developed by using 222 microsatellite markers. The previous female and male map consisted of 25 and 23 linkage groups with 67.2 and 67.8 % of genome coverage and 1.5:1 of average recombination ratio (Liu et al. 2013). In the new genetic linkage map, 714 SSR markers were mapped in the 24 linkage groups, which is consistent with the diploid chromosome number of the kelp grouper (2*N* = 48) (Lan 2009). About 509 and 512 markers were identified and evenly covered the 24 linkage groups of the female and male maps, respectively. Only 10 of 714 markers remained as single markers. All of the microsatellite markers used in the previous genetic linkage map were also included and were consistently assigned in the same order and linkage groups in the present study, except for six markers. Of these, three markers (EguStr125DB, MiniSTR267DB, and Ebr00025FRA) and three other markers (MiniSTR266DB, Ebr00270FRA, and Ebr00253FRA) in linkage group EBR 24 and EBR 25 of the female map were moved to linkage groups EBR 23 and EBR 5 in the new female map, respectively. In addition, the genome coverage and average ratio of recombination between female and male maps were about 84.68, 83.21, and 1.12:1, respectively. This result revealed a large number of markers in the F_1_ progeny that filled several gaps of the new linkage map, which led to a reduction in the average mapping interval and an increase of the genome coverage. Considering the average interval and the genome coverage, we conclude that the high-resolution genetic linkage map of the kelp grouper of this study offers a sufficient marker density to permit a preliminary genome-wide scan for QTLs for growth-related traits (Massault et al. 2008). In addition, markers from other grouper species could speed up the construction and completion of a genetic linkage map of the kelp grouper in the near future.

The recombination rate of a gene located on a chromosome (autosomal) is different between females and males because of the number of crossing-over events that occur during meiosis I. Differences in recombination rates between sexes have been identified in many species; for example, humans (Dib et al. 1996), dogs (Wong et al. 2010), crocodiles (Miles et al. 2009), and fish. In fish, recombination rates have generally been reported to be higher in females compared to males ranging from 3.25:1 in rainbow trout (Sakamoto et al. 2000), 7.4:1 in the Japanese flounder (Coimbra et al. 2003), 1.37:1 in Atlantic salmon (Lien et al. 2011), 2.2:1 in the silver carp (Guo et al. 2013), 2:1 in the Atlantic halibut (Reid et al. 2007), 1.5:1 in the kelp grouper (Liu et al. 2013), 1.03:1 in the orange-spotted grouper (You et al. 2013), and 1.19:1 in the white grouper (Dor et al. 2014). In this study, the recombination rate ratio between females and males was 1.12:1, which was lower than previous reports. This may reflect the increased number of markers linked to the male map rather than the female map, which would affect not only the density of the markers but also the recombination rate in all linkage groups. In the present study, we found that markers in the female and male maps were irregularly distributed and showed high clustering of markers in all linkage groups. These markers tended to be compressed in the telomeric and centromeric regions of the female and male maps. A higher rate of recombination in the female and male maps probably occurred near the centromeric and the telomeric regions (You et al. 2013). This could be explained by the higher frequency of recombination in females near the centromeric regions during oogenesis. Similarly, more frequent recombination in males was also found near the telomeres during meiosis (Strachan and Read 2011; You et al. 2013). For indicating the centromeric or telomeric region in female and male maps, these two regions were observed by the map distance between markers. In the case of high recombination, the maps will present high distance between markers or clusters. The distances between markers in the centrometric region were assessed to be larger than other sites (telemetric). Similar to the male map, the markers or clusters in telemetric regions were estimated to have a larger distance than the centrometric region. The difference in sex recombination is an important factor in the implementation of marker-assisted selection using QTL-associated mapping.

The growth-related quantitative trait QTLs in this study were identified using F_1_ progeny of the kelp grouper. This was different from other studies that performed QTL mapping using F_2_ generation from F_1_ crosses in a genetically different line or F_2_ back-cross (Hayashi and Awata 2004), such as the Pacific white leg shrimp (Andriantahina et al. 2013). Kelp groupers are protogynous hermaphrodites and it would take a long time to produce an F_2_ generation. This type of reproductive system takes a longer time for the sex reversal from male to female when they exceed a certain age or body size. In the kelp grouper, it takes more than 6 years of culture for the fish to reach maturity (before the first maturation and spawning). This is too long to create an F_2_ generation. This explains our choice of producing F_1_ progeny for the QTL study. In the past decade, the analysis of QTLs using F_1_ progeny was developed and successfully applied to Asian seabass (Wang et al. 2006). Under the criteria of heritability of traits of interest, the power of QTL detection depends on the heritability of the traits, the effect of alleles involved, the recombination distance of the associated marker, and the sample size (Mackay 1996). We found a major QTL affecting BW in the kelp grouper that was located on linkage group EBR 17F of the female map under genome-wide linkage analysis. We also found putative QTLs affecting BW that were located in seven linkage groups under a chromosome-wide analysis. The phenotypic variance of the major QTL was 14.6–18.9 and was 7.5–12 % for the putative QTLs. Similar results were obtained for the total length trait. One major QTL was detected in the same linkage group of BW that explained 14.7–18.5 % of the phenotypic variance. The putative QTLs accounted for 7–11.3 % of the phenotypic variance. These results indicated that several QTL region-associated BW and TL traits are determined by multiple genes. Our result also revealed that the growth-related traits of the kelp grouper might be controlled by a few QTLs with large effects.

The candidate QTLs were confirmed in two developmental stages in families A and B, with 35 representative markers. The results showed a highly significant level for major QTL in stage II of family A after adding the number of progeny, which were rejected in stage I of family A and both stages of family B. For the putative QTL regions in stage II of family A on linkage groups EBR 13F and EBR 18M, the results were rejected for stage I family A and stage II of family B. However, they were accepted for stage II family A and stage I family B with same regions on linkage group EBR 17M of the male map (qBW17m-1). From these results, we considered that the explanation lay in the parental fish, the distribution of the phenotype, and the number of progeny. In addition, we noticed the significance of the LOD score of the candidate major QTL decreased after confirmation of the significant QTL region, while the LOD of the putative QTL region increased. This was particularly true for the putative QTL affecting BW and TL on linkage group EBR 13F after we increased the number of progeny. It is possible that given a sufficiently large number of progeny, more major QTL regions could be detected and confirmed.

Herein, the most important finding was a single peak of QTL associated with BW and TL within the proximal region of linkage group EBR 17F. Both QTL (qBW17f and qTL17f) peaks were located at position 22.4 cM, with 99 % confidence interval mapping within 4.4 cM of the most proximal markers from Ebr00702FRA to Ebr00092FRA by simple interval mapping. The narrowness of the interval marker of the candidate QTL region should be considered as a fine approximation, given the large QTL effect and high recombination rate found in kelp grouper females. These results could be used to investigate candidate genes in a future study of growth-related traits of the kelp grouper.

## Conclusions

This study constructed the first high-resolution genetic linkage map of the kelp grouper. The map provided an increased SSR marker density from 222 microsatellite markers on the first-generation genetic linkage map (Liu et al. 2013) to 716 SSR markers. Twenty-four linkage groups were identified, consistent with the 24 haploid chromosome number of the kelp grouper (2*N* = 48). The female and male maps accounted for 84.68 and 83.21 % coverage and produced average mapping intervals of 4.1 and 4.0, respectively. Considering the average mapping interval and genome covered, these linkage maps would be sufficient for genome-wide linkage analysis and could increase the power of statistics to detect growth-related QTL traits.

Three significant QTLs affecting both phenotypes (BW and TL) were detected and confirmed. One major QTL was significant (1 and 5 % at the experiment-wide significance level) in linkage group EBR 17F of the female map, which showed 6–8.6 and 6.1–8.8 % of the phenotypic variance. Two putative QTLs affecting both phenotypes (BW and TL) (5 % chromosome-wide significance level) were located on linkage groups EBR 13F and EBR 18M of the female and male maps, explaining 1.8–9.1 and 1.2–8.7 % of the phenotypic variance. These results suggested that the growth-related quantitative traits are controlled by multiple genes.

We anticipate that the high resolution of genetic linkage map and growth-related QTLs found in this study could be applied to find candidate genes, will be powerful tools for a future MAS breeding program and may provide further insights into the genetic control of growth traits in the kelp grouper.

## Electronic supplementary material

Additional file 1 Normal distribution and Pearson correlation coefficients of total length and body weight in stage I and II of families A and B. **a** Distribution of total length and body weight and Pearson correlation coefficients between the total length and body weight in stage I of family A (360 progeny). **b** Distribution of total length and body weight and Pearson correlation coefficients between the total length and body weight in stage II of family A (163 progeny). **c** Distribution of total length and body weight and Pearson correlation coefficients between the total length and body weight in stage I of family B (112 progeny). **d** Distribution of total length and body weight and Pearson correlation coefficients between the total length and body weight in stage I of family B (45 progeny). (JPEG 921 bytes)

High Resolution Image (TIFF 970 kb)

Additional file 2 List of SSR markers used to construct a high-resolution genetic linkage map of the kelp grouper. EBR markers were developed using microsatellite-enriched segments from next-generation sequencing (NGS) using the GS FLX system (Roche, Switzerland); STR markers were developed using simple sequence repeats (SSRs) of a cross species of grouper from the NCBI database. (XLSX 204 kb)

Additional file 3 List of SSR markers used to confirm the QTL regions of the kelp grouper. EBR markers were developed using microsatellite-enriched segments from next generation sequencing (NGS) using the GS FLX system (Roche, Switzerland); STR markers were developed using simple sequence repeats (SSRs) of a cross species of grouper from the NCBI database. (XLSX 23 kb)
