# Supplementary figures and images for: Detection of Growth-Related Quantitative Trait Loci and High-Resolution Genetic Linkage Maps Using Simple Sequence Repeat Markers in the Kelp Grouper (Epinephelus bruneus)
Source: Mar Biotechnol (NY). 2015 Oct 28;18:57–84. doi: 10.1007/s10126-015-9673-5 (PMC4705122; doi:10.1007/s10126-015-9673-5)

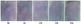

Supplement: Supplementary file 1 — Normal distribution and Pearson correlation coefficients of total length and body weight in stage I and II of families A and B. a Distribution of total length and body weight and Pearson correlation coefficients between the total length and body weight in stage I of family A (360 progeny). b Distribution of total length and body weight and Pearson correlation coefficients between the total length and body weight in stage II of family A (163 progeny). c Distribution of total length and body weight and Pearson correlation coefficients between the total length and body weight in stage I of family B (112 progeny). d Distribution of total length and body weight and Pearson correlation coefficients between the total length and body weight in stage I of family B (45 progeny). (JPEG 921 bytes) [file 10126_2015_9673_Fig6_ESM.jpg]

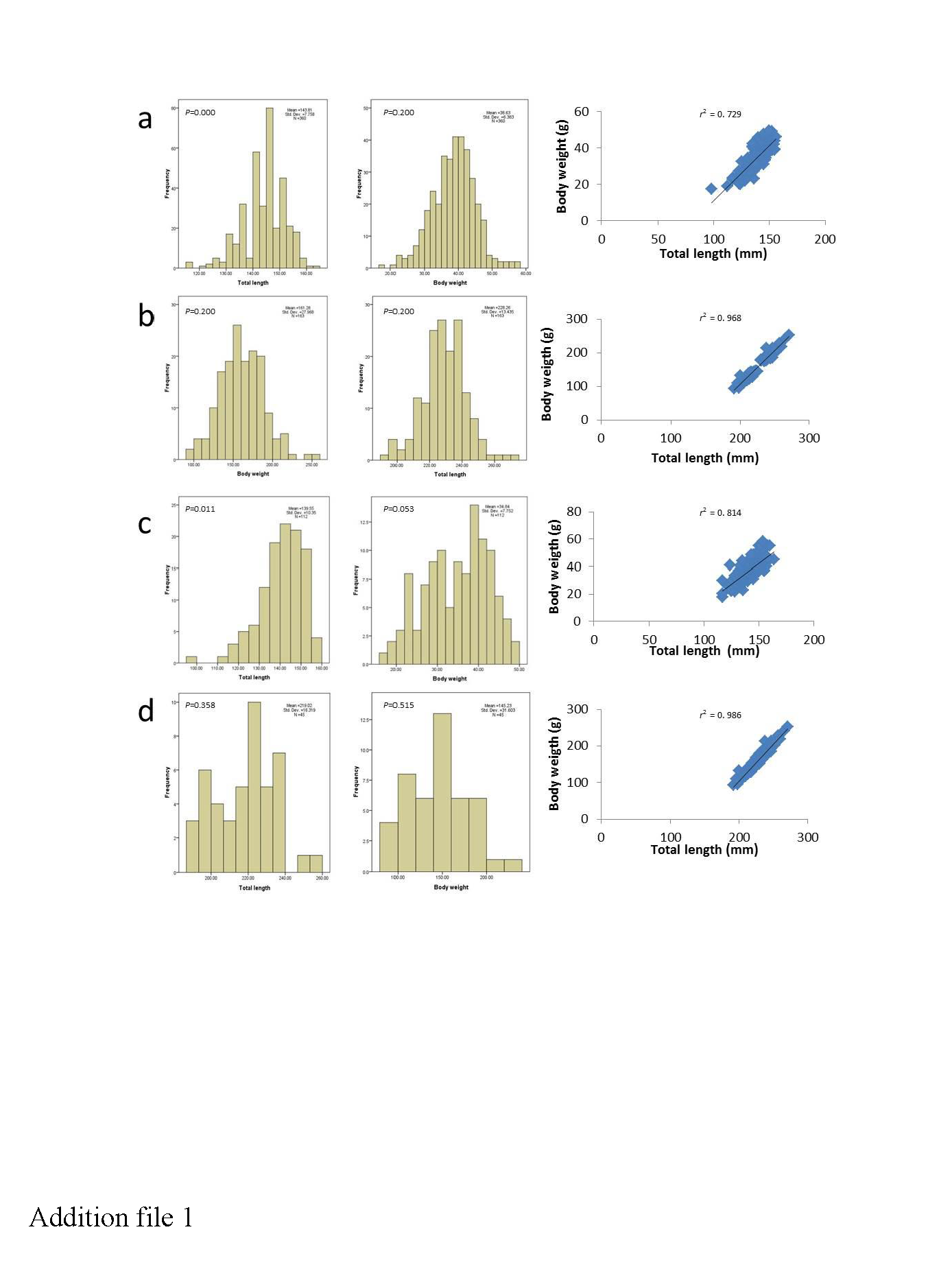

Supplement: Supplementary file 2 — High Resolution Image (TIFF 970 kb) [file 10126_2015_9673_MOESM1_ESM.tiff]
